# Sampling frequency matters: mapping of the healthy infants' gut microbiome during the first year of life

**DOI:** 10.1016/j.crmicr.2025.100470

**Published:** 2025-09-09

**Authors:** Lenka Micenková, Soňa Smetanová, Jacek Marciniak, Kristýna Brodíková, Dominika Polaštík Kleknerová, Barbora Lakotová, Barbora Zwinsová, Vojtěch Thon, Petra Vídeňská, Eva Budinská

**Affiliations:** a RECETOX, Faculty of Science, Masaryk University, Kamenice 5, 625 00 Brno, Czech Republic; b Department of Experimental Biology, Faculty of Science, Masaryk University, Kotlářská 2, 611 37 Brno, Czech Republic

**Keywords:** Infant, Newborn, Gut, Microbiome, Sequencing, Sampling

## Abstract

Early life events significantly influence the developing gut microbiome, yet the response time and duration of microbiome changes to specific factors, such as vaccination or solid food introduction, remain unclear. Consequently, determining the optimal sampling frequency to monitor gut microbiome development is challenging. This study monitored gut microbiome plasticity using 16S rRNA gene sequencing almost daily in one infant (A) and weekly in 12 others (B–M) during their first year. Changes were linked to external factors and their duration analyzed. Three bacterial colonization groups emerged: "Early-life colonizers," "Re-appearing colonizers," and "Later-colonizers." Weekly sampling underestimated microbiome variability, as individual samples within the same week differed by over 1 Shannon index, and most of the weekly coefficients of variation of different alpha diversity indices in the first 23 weeks were higher than 10 %. Alpha diversity variability decreased with age, but beta diversity variability remained high. Key events like solid food introduction and probiotics caused gradual but significant bacterial composition changes, with effects varying among infants. Sparse weekly sampling hindered a detailed understanding of the impact of maternal microbiome, diet, probiotics, vaccinations, and unforeseen variables. Analysis of weekly variability in alpha and beta diversity suggests that such rare sampling may not be sufficient in terms of the outcomes of interest.

## Highlights

• Gut microbiome monitored daily in 1 infant and weekly in 12 during the first year.
• Three bacterial groups emerged: Early-life, Re-appearing, and Later colonizers.
• Alpha diversity variability decreased with age, but beta diversity remained high.
• Solid food, vaccination, and probiotics caused significant, individualized shifts.
• Downsampling analysis showed that weekly sampling misses transient changes.

## Graphical abstract

**Figure:**
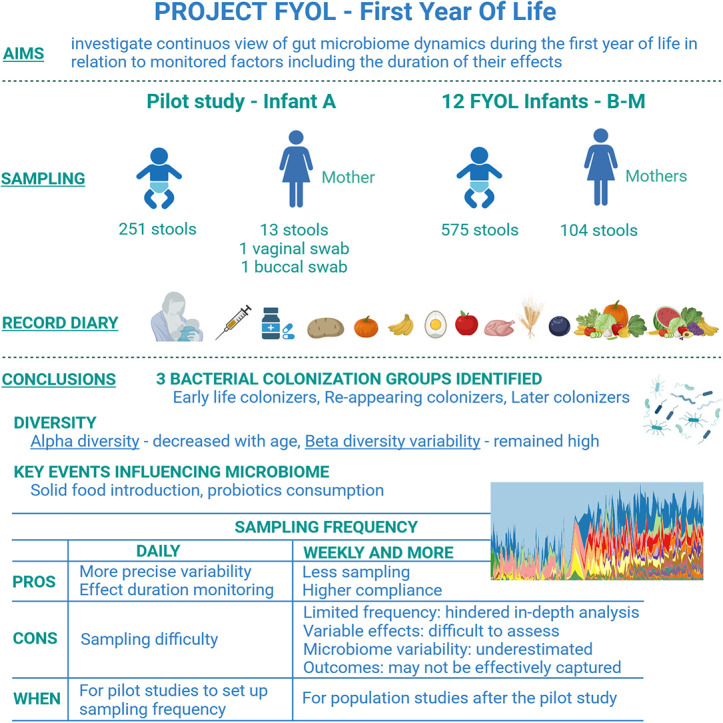

## Introduction

The first year of life is a critical period of early childhood growth and development marked by rapid maturation of metabolic, endocrine, neural, and immune systems. An interesting perspective on infant developmental biology includes trillions of microbes that reside within the body and are involved in many aspects of human health. The composition of the intestinal microbiome acquired in infancy has been proven to be a crucial factor for child health in later life. The alterations of gut microbiota and low bacterial diversity seem to play a role in different pathological conditions like inflammation, atopic eczema, obesity, increased risk of asthma, and allergies in childhood and adulthood (Jong, 2014; Tamburini et al., 2016; Robertson et al., 2019). Research on early-life gut microbiota colonization is still ongoing, but it is clear that this process is influenced by factors such as delivery type, illness, diet, medication, probiotics, and toxin exposure. Most studies collect samples at specific time points, either based on developmental milestones or without clearly defined criteria, with sampling typically occurring between one and 24 times per year, often within the first week of life and at the 1st, 6th, 9th, and 12th months. Some studies on evolutionary dynamics have just prospectively collected samples from different children and categorized them by age (Rodríguez et al., 2015; Bokulich et al., 2016; Nuriel-Ohayon et al., 2016; Zhong et al., 2019; Stanislawski et al., 2018; Palmer et al., 2007; Bergström et al., 2014; Putignani et al., 2014; Nagpal and Yamashiro, 2018; ; ;Wampach et al., 2017; [Bibr bib0064][Bibr bib0053] Gritz and Bhandari, 2015; Vandenplas et al., 2020; Suárez-Martínez et al., 2023; Niu et al., 2020). However, these approaches may miss the inherent variability within an individual's microbiome and fail to capture its plasticity in response to early-life events, and the duration of microbial changes after each event.

Because such high-resolution longitudinal data are almost never available in infancy, we aimed to generate a quasi-daily time series in one infant to explore what biological signals might be lost with conventional sparse sampling.

In our unique study, we collected 251 fecal samples from a single infant during the first year of life and analyzed them using 16S rRNA gene sequencing to provide a continuous view of gut microbiome dynamics in relation to monitored factors and events, including the duration of their effects. We also analyzed the mother's intestinal, oral, and vaginal microbiomes as key sources of bacterial colonization. An additional set of twelve infants and their mothers were sampled weekly and monthly (575 stool samples from infants, and 104 from mothers). This dataset was used for assessing whether the changes identified in the almost-daily sampled infant A could also be observed with a more scarce sampling approach.

## Methods

**Fig. 1:**
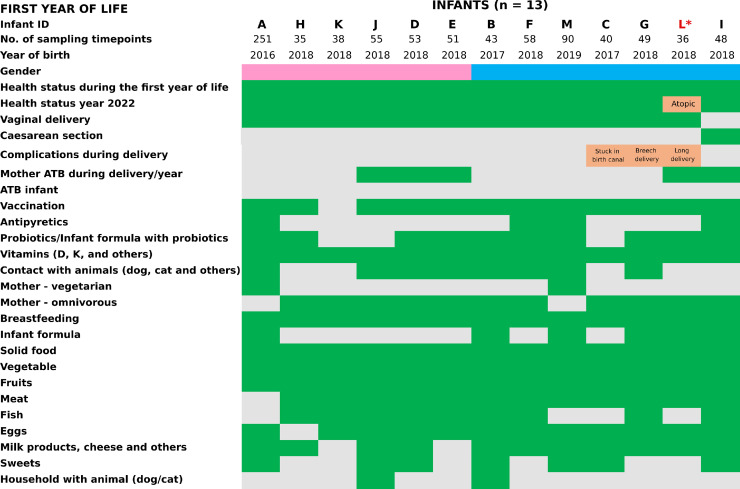
**Key information on the infants (*****n*****= 13) sampled during the first year of life.** "Yes" responses are indicated in green and "No" responses are in grey. Female gender is represented in pink and male gender in blue. *Infant L was diagnosed with atopic eczema at the beginning of the second year of life, therefore, this child's results were excluded from the statistics for the other healthy children.

The study design is summarized in Fig. S1. All samples were collected prospectively by the mothers, who were provided with collection kits and detailed instructions. The optimal sampling frequency was set as daily for infant A and weekly for infants B-M, though mothers were not always able to adhere to the schedule. For infant A, who was sampled almost daily, 251 meconium/stool samples from the infant and 15 samples from the mother (13 fecal, 1 buccal, and 1 vaginal) were collected during the first year. Mother A, who followed a vegetarian diet for over 20 years, also served as the corresponding author and principal investigator. She and her infant were selected for near-daily sampling due to the high feasibility of maintaining strict adherence to sampling protocols over one year. Both mother and infant followed a vegetarian diet during the study period, and maternal blood markers were monitored regularly to confirm adequate nutritional status. For the other 12 infants (B-M), who were sampled approximately weekly, 575 meconium/stool samples from the infants and 104 stool samples from their mothers were collected. Detailed information on the sampled infants is provided in Fig. 1 and Table S1. There were no significant differences in race/ethnicity, household income, housing, or parental education level among the subject families. All participating families were of Caucasian ethnicity and shared a comparable socioeconomic background, including similar household income levels and stable housing conditions. All parents held a university-level education. This demographic homogeneity was maintained to reduce potential confounding variables related to socioeconomic status or educational attainment. None of the infants attended daycare during the first year of life. All were cared for at home by their parents. Three infants (H, K, and M) had older siblings (with age gaps ranging from 2 to 5 years), while the remaining infants were first-borns. The effect of sibling presence was not formally analyzed due to limited statistical power but may represent a factor worth investigating in larger studies.

A stool container (FL Medical, Italy) was used to collect the mother's stool, and a flocked swab (Copan, Italy) was used for the collection of infant meconium/stool/oral samples or mothers' vaginal smear. All samples were stored in domestic freezers at −20 °C until collection. Samples were picked up by study staff directly from participants' homes and transported to the laboratory on dry ice to ensure temperature stability. Sample collection and transport were performed most frequently in three batches over the course of the year.

### DNA isolation from samples

The DNeasy PowerLyzer® PowerSoil® Kit (QIAGEN, Germany) was used to extract DNA from all sample types. After thawing, the mother's fecal sample in the stool container was diluted 5-fold with molecular-grade water and homogenized by vortexing with 2.3 mm Zirconia beads (BioSpec, USA). A volume of 260 µl of homogenized stool was then used for DNA isolation according to the manufacturer's instructions. Flocked swabs containing the infant's meconium, fecal, or oral samples, as well as mothers' vaginal smear were transferred into 2 ml tubes for subsequent DNA extraction. To the swab samples, 750 µl of Bead Solution and 60 µl of C1 Solution were added, followed by thorough vortexing and centrifugation. The swabs were then removed, and the samples were homogenized using the FastPrep-24 (MP Biomedicals, USA) for 45 sec at 6.5 m/s. The supernatant was removed from the centrifuged samples, and DNA extraction was then performed according to the manufacturer's protocols. The extracted DNA was stored in an elution buffer at −20 °C.

### PCR amplification and Illumina library preparation

The isolated DNA was used as a template for PCR reactions targeting the hypervariable V4 region (EMP 515–806) of the bacterial 16S rRNA gene, following the 16S Metagenomic Sequencing Library Preparation protocol (Illumina, San Diego, CA) with minor deviations, as described in Videnska et al. (2019) (Table S2). Sequencing was then performed using MiSeq Reagent Kits v2 on a MiSeq 2000 sequencer, in accordance with the manufacturer's instructions (Illumina, USA).

### Bioinformatic data analysis

Raw sequence reads were quality-filtered and demultiplexed using a custom preprocessing pipeline implemented in Bash and Python. No novel algorithms were introduced; all steps used well-established tools. The code is available from the authors upon request. This in-house pipeline automated standard 16S amplicon processing steps, including demultiplexing based on dual barcodes using pattern matching, quality trimming using Trimmomatic (v0.39), length filtering and adapter cropping. Following preprocessing, forward and reverse reads were denoised using the DADA2 amplicon denoising R package (Callahan et al., 2016). Following denoising, the forward and reverse reads were joined using join_paired_ends.py from QIIME v1.9.1 the fastq-join read joining utility (Caporaso et al., 2010).

Finally, chimeric sequences were removed from the joined reads using the removeBimera function of the DADA2 R package. The taxonomy was determined using the Usearch-consensus algorithm from the microbiome analysis toolkit QIIME (v 1.9.1) (Caporaso et al., 2010). For each input sequence, the three closest organisms were found in the Silva v.123 reference database (Quast et al., 2013). Their taxonomies were combined into the final taxonomic assignment using the least common ancestor (LCA) algorithm. Taxonomic names of bacterial phyla obtained from the Silva database were corrected according to the publication by Oren and Garrity (2021).

### Statistical analysis

Statistical analysis of microbiome data was done at the phylum and genera level, and the diversity of the microbial community was estimated by the Shannon diversity index. Data were treated as compositional and prior to all statistical analyses, were transformed using centered log-ratio (CLR) transformation (Aitchison, 1986). All zeroes in the original data were replaced using the count zero multiplicative method (Martin-Fernandez et al., 2015). Only genera that were detected in at least 2 % of samples with a minimum abundance of 10 reads were included in the transformation process and additional statistical analysis of the microbiome to avoid high sparsity in data.

Unsupervised homogeneous microbial diversity analyses were performed to divide each infant's stool samples into various numbers of periods with homogeneous diversity of the infant's gut microbial community. Automated breakpoint detection analysis (circular binary segmentation) was used - the significance level for the test to accept change-points was set to 0.01, and the minimum number of markers for a changed segment was 2 (Olshen et al., 2004; Venkatraman and Olshen, 2007). The meconium sample (originally in period 1) was settled as period 0 to emphasize it as a different stool type.

For infant A, microbial compositions in the 7-day period before and after the occurrence of an event (medication administration, vaccination, nutritional supplement administration, etc.) were compared by the Mann–Whitney U test. To test changes before, during and after longer-lasting events, such as first period of probiotic consumption and type of nutrition (breastfeeding vs. solid food), Kruskal-Wallis test by ranks with Dunn's post hoc test was used. To evaluate the effects of early-life events (e.g., dietary transitions, vaccinations, probiotic intake) in infants B–M, we applied non-parametric statistical tests tailored to each context. For group-level events such as the introduction of solid foods or routine vaccinations, we used the Friedman test with Conover post hoc correction. If multiple samples were available per period for a given infant, bacterial genera abundances were averaged to yield a single representative profile per time window. These samples were chosen to minimize the time interval between them - typically within 7–10 days - to reduce the impact of age-related microbiome drift. No standardized age window was applied across infants, as events occurred at varying developmental stages. This approach preserves the within-subject temporal structure while enabling group-level comparison across events, without inferring between-subject generalizability.

The analytical windows for all tests were defined as follows:

- **Vaccinations:** Due to limited temporal resolution (exact vaccination day was often not recorded), we compared microbial profiles across the week prior, the vaccination week, and the week after.
- **Dietary transitions (solid food):** Changes in diet were tested across five 28-day intervals - the final month before solid food introduction and four subsequent monthly intervals - capturing progression through complementary feeding.
- **Probiotic supplementation:** As probiotic administration was variable and limited to specific individuals, we used the Kruskal-Wallis test at the individual level, comparing microbiome composition max 30 days before, during, and 30 days after probiotic exposure.

Infant A received Espumisan (simethicone) intermittently during the early months due to symptoms of colic. The medication is non-systemic and not known to alter the microbiome, but its use is documented for transparency. No other infants in the cohort received Espumisan regularly.

The resulting *P*-values were adjusted for multiple hypothesis testing using the Benjamini–Hochberg procedure (function *p.adjust* in R package *stats*). Results were considered significant at *FDR ≤ 0.1.*

To test the influence of age and individuals (infants B-M) on microbial composition, PERMANOVA on Euclidean distances of CLR-normalized data (999 permutations) was used.

To assess the effect of sampling frequency on microbial composition and statistical significance of tests, we performed a downsampling analysis of infant A's high-resolution dataset. One sample per week (*n* = 52) was randomly selected to simulate weekly sampling. This downsampling was repeated five times, and care was taken to select different samples for each iteration when possible. For each genus, we then calculated how many weeks it was detected in each downsampling run. Statistical analyses for the effects of early-life events were performed as described above, except for vaccination, where three samples representing approximately three weeks prior to and three weeks following the first dose were used for statistical testing. This wider interval was selected in contrast to the previously used 7-day window, as a minimum of three observations per group was required to enable valid statistical comparisons.

All statistical analyses were performed in R, version 4.0.3 (R Core Team 2020) using additional R packages zCompositions (zero replacement, ver. 1.4.0–1), compositions (CLR transformation; ver. 1.40–3), PSCBS (unsupervised breakpoint analysis; ver. 0.66.0), gplots (heat maps; ver. 3.0.1.1), factoextra (PCA plots; ver. 1.0.5) and ggplot2 (area plots, bar plots, timeline charts; ver. 3.3.3) and UpSetR (visualization of bacteria intersections in different sample matrices) (Martin-Fernandez et al., 2015; van den Boogaart et al., 2019; R Core Team R, 2019; Palarea-Albaladejo and Martin-Fernandez, 2015; Kassambara and Mundt, 2017; Warnes et al., 2019; Wickham, 2016; Gehlenborg, 2019).

## Results

For all infants, mother-maintained diaries tracking health, child development, medication, vaccination, breastfeeding, solid food introduction, probiotic use, and environmental factors like household pets. No infant took antibiotics during the first year of life. Infant L was diagnosed with atopic eczema at the beginning of the second year of life, therefore, this child's results were excluded from the statistics for the other healthy children (Fig. 1).

We present the results at the bacterial phylum and genus taxonomic levels. Taxonomy was assigned down to the genus-equivalent level; clusters without sequences matching known genera were named according to the lowest possible taxonomic level.

To assess changes in the alpha diversity and bacterial composition of fecal microbiota, we employed supervised and unsupervised approaches. The unsupervised approach consisted of automatic breakpoint detection in the time-ordered Shannon index, which identified homogeneous time periods with consistent alpha diversity. Additionally, we performed hierarchical clustering of the bacterial abundances to identify groups of bacteria with similar patterns of abundance in time. In the supervised approach, we performed statistical testing of changes in alpha-diversity or abundance of selected bacterial genera before vs. after the monitored event (see Methods). The combination of both types of analyses provided comprehensive information about the onset and the duration of the effects of the monitored variables.

### Colonization phases and reactions of the fecal microbiome of the first year of life

**Fig. 2:**
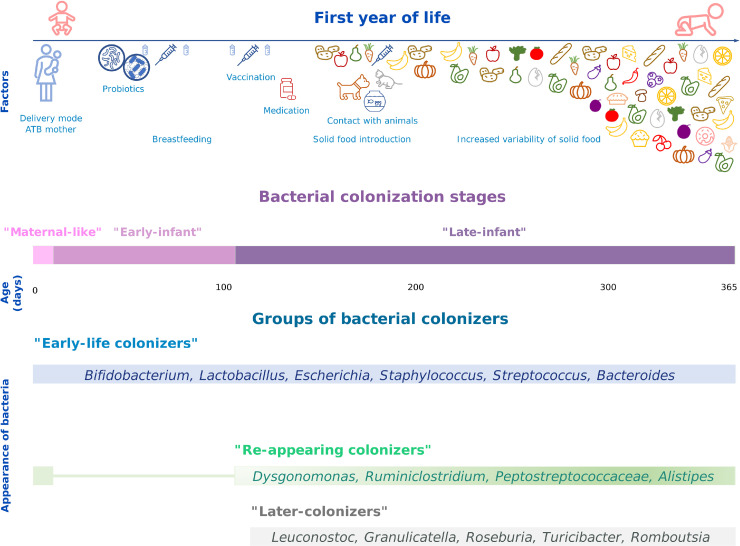
**General scheme of stages of bacterial colonization and groups of bacterial colonizers.** Selected bacterial colonizers during the first year of life of almost daily monitored infant A.

**Fig. 3:**
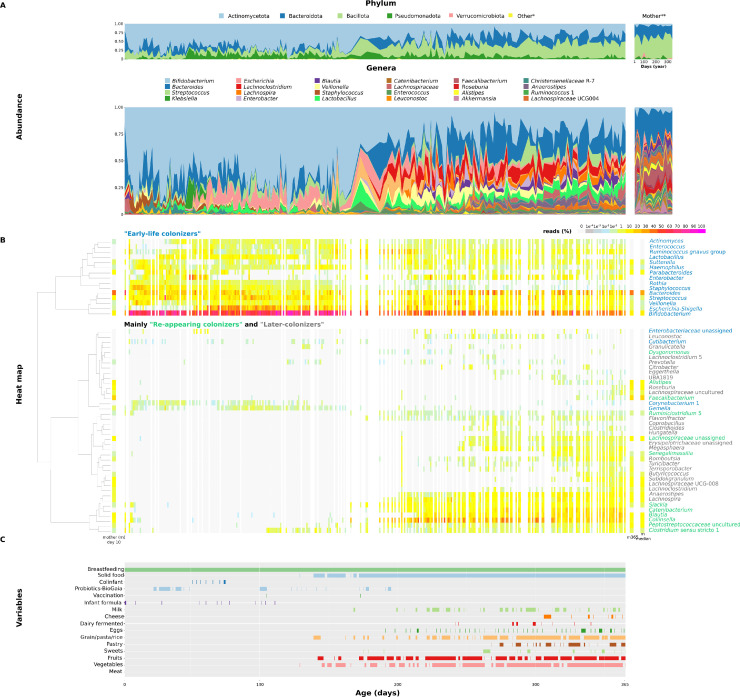
**Composition of infant A's and mother's fecal microbiome during the first year of life. A.** Dynamics of the fecal microbiome at the phylum or genus level. *Bacteria with a maximal incidence below 1 %. **Mother's stool samples collected throughout the year. **B.** Heatmap dendrogram showing the co-occurrence of 55 bacterial genera (genera present in at least 2 % of samples with a minimum abundance of 10 reads) in time. Clustering was made using Ward's method on Euclidean distances of CLR-normalized bacterial abundance data. White columns indicate missing samples. **C.** Main factors/variables/events monitored during the year.

In the almost daily monitored infant (infant A), we tracked the dynamics of bacterial phyla and genera (Fig. 3A), identifying three groups of bacterial colonizers with similar abundance patterns. The first stage of colonization, influenced by the mother's microbiome, was termed "Maternal-like" stage (first 1–2 weeks of life). Two main groups of colonizers emerged - "Early-life colonizers", which were established quickly and persisted throughout the year, and "Re-appearing colonizers", detected immediately after birth, then disappearing and re-emerging alongside the third group, "Later-colonizers". The dominance of "Early-life colonizers" defined the "Early-infant" colonization stage. The subsequent most diverse period, where all three colonizer groups coexisted, was labeled the "Late-infant" stage (Fig. 2).

In Fig. 3B, the detailed appearance of bacterial colonizers summarized in Fig. 2 is shown. The most diverse stage where all three groups ("Early-life colonizers", "Re-appearing colonizers", and "Later-colonizers") were present, coincided with the introduction of solid foods (Fig. 3C).

In infant A, the meconium sample showed no detectable bacterial population, but subsequent stool samples revealed ten different phyla, 99.8 % of which were represented by Actinomycetota, Bacillota, Bacteroidota, and Pseudomonadota (Fig. 3A). Only three genera were detected in at least 99 % of the 250 stool samples collected throughout the year: *Bifidobacterium* (median 48.6 %), *Bacteroides* (median 7.1 %), and *Streptococcus* (median 3.1 %). Additionally, *Escherichia*-*Shigella* (median 3.9 %) and *Veillonella* (median 1.7 %) were among the most dominant genera (i.e., genera with a median > 1 % across the year)*.*

The transient reduction in *Bacteroides* and concurrent increase in *Bifidobacterium* observed at day 98 in mother A stool sample may reflect technical variability rather than a sustained biological change, as supported by the atypical profile of this single sample.

**Fig. 4:**
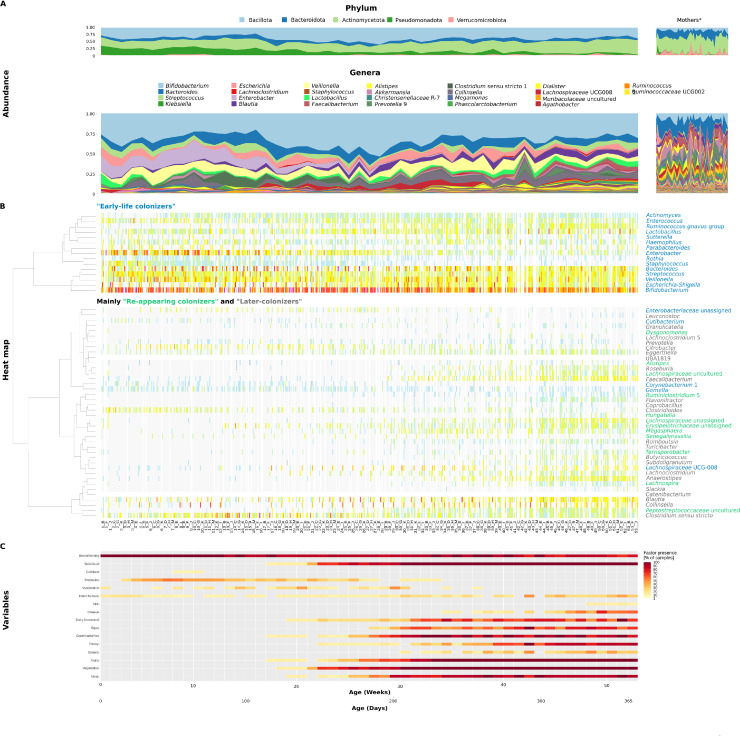
**Composition of the infants (B-K and M) and mothers fecal microbiome during the first year of life. A.** Dynamics of the fecal microbiome at the phylum or the genus level. Average weekly relative abundances of all infants and mothers were used. Genera are ordered mainly by infant A (Fig. 3A); less abundant genera in infants B–K and M are omitted in color legend, and more abundant unique genera are shown there instead in Fig. 4A. *Mothers stool samples collected during the year. **B.** Heatmap showing the co-occurrence of 55 bacterial genera in time - bacterial colonizers of infants B-K and M were ordered based on the clustering from infant A (Fig. 3B). All samples were used. White indicates missing values for the given week and child. For better readability and to clearly indicate the week from which each sample comes, only a selection of labels is shown in the heatmap. **C.** Main factors/variables monitored during the year.

Next, we aimed to determine whether the weekly sampling of infants B-M could capture or validate the bacterial colonization patterns observed in the near-daily sampling of infant A. For this, we reconstructed the infant A heatmap (Fig. 3B) such that we aligned all the bacterial compositions of samples of infants B-K and M together on a relative timeline from birth to 1 year and ordered the bacteria based on the clustering dendrogram from infant A (Fig. 4). Similar to infant A, *Bifidobacterium* was the most prevalent genus, detected in nearly all samples from all infants. This bacterium was part of the group of "Early-life colonizers" along with other genera like *Staphylococcus, Streptococcus*, and *Bacteroides*, which were easily recognized also in weekly sampling (Fig. 4B). Similarly, "Later-colonizers" bacteria groups over time were recognized within weekly sampling (Figs. 3B and 4B). The occurrence of various "Later-colonizers", as in infant A, was also primarily linked to early life events or factors (Fig. 4C). Some of the "Re-appearing colonizers" which were detected immediately after birth then disappeared and reappeared were more difficult to spot within weekly sampling, due to the fact that their period of first appearance was very short (1–2 weeks) and this can be confused in weekly sampling for an artifact, compared to the daily sampled infant A, with their most often appearance (e.g., *Ruminiclostridium* 5). Given that this pattern was observed only in infant A with near-daily sampling and not consistently across the cohort, we interpret the "Re-appearing colonizers" as a hypothesis-generating observation rather than a broadly generalizable category. A PCA plot of infant A's bacterial composition at the genus level revealed age-related variability (Fig. S2A), this pattern was observed across all infants (Fig. S2B). The PCA plot and PERMANOVA of the 11 weekly sampled infants indicated that both age and individual infants (Fig. S2C) had a statistically significant influence on bacterial composition (BH/adjusted q-value = 0.001).

### Identification of unsupervised homogenous microbial diversity time periods

**Fig. 5:**
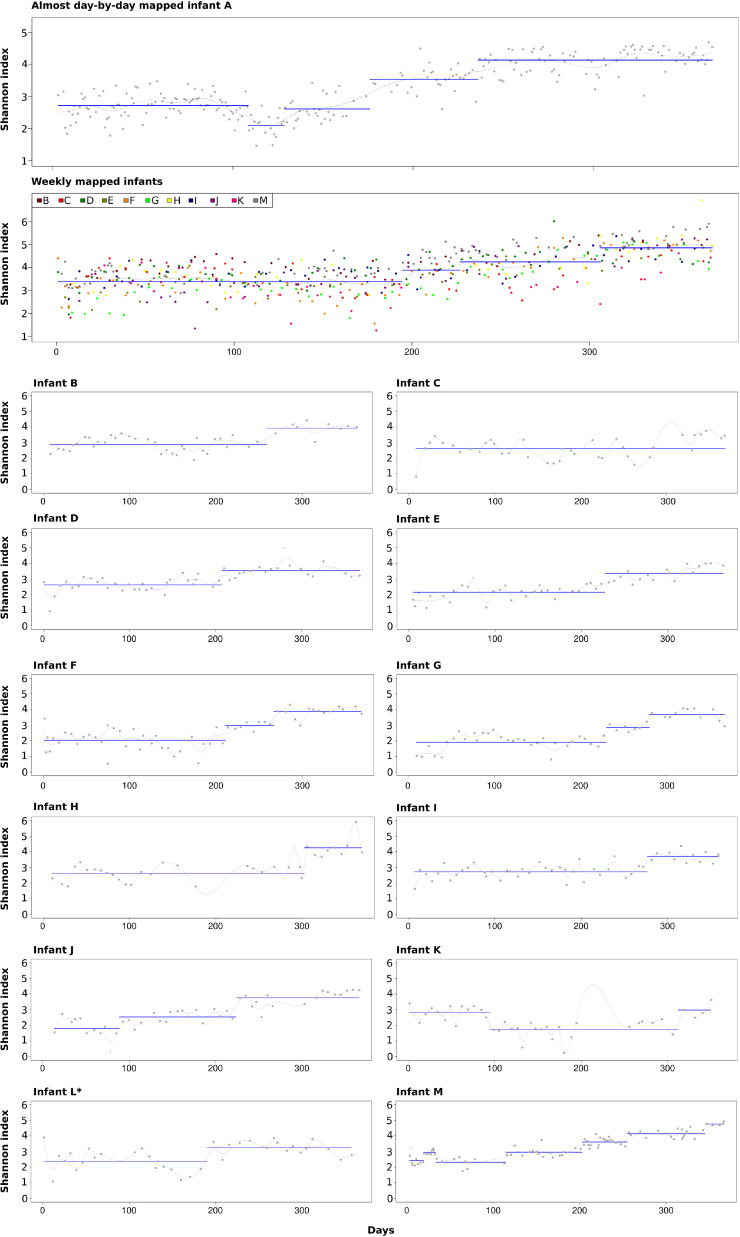
**Homogeneous Microbial Diversity Periods (according to the Shannon diversity index) identified in infants during the First Year of Life.** *Infant L, diagnosed with atopic eczema at the start of the second year, was excluded from the general figure of weekly sampled infants.

In all infants, microbial diversity measured by the Shannon diversity index and the number of identified bacteria increased from the neonatal period to the end of the year. In infant A, unsupervised automated breakpoint detection identified five statistically homogeneous microbial diversity (HMD) periods (Fig. 5). As expected, diversity gradually increased over the year, but especially after the introduction of the solid food, there was a gradual plateauing, culminating in the last stable period with the highest diversity lasting >100 days, confirming the establishment of the microbiome. Certain monitored life events aligned with specific HMD periods, such as the emergence of a new period after vaccination (HMD period 2). HMD period 1 was mainly linked to breastfeeding and HMD period 5 was related to the high solid food intake.

In samples from infant M (sampled more frequently than most, but less than infant A), more HMD periods were identified, similar to infant A. In other children, likely due to sparse sampling, we could only identify approximately two major HMD periods, and four periods when combining data from infants B-K and M (Fig. 5). These sampling limitations hindered a full understanding of microbiome dynamics, highlighting the need for denser, more regular sampling in future studies. Nonetheless, identifying these two periods offers valuable insights into the early stages of gut microbiome development. In the next section, we analyze the potential factors influencing microbiota changes and the emergence of HMD periods.

### Approaching the mothers' gut bacterial composition

To consider possible sources contributing to the composition of the infant's A early gut microbiome, samples were collected from the mother's buccal and vaginal swabs and stool around the time of delivery. An UpSet plot was used to visualize intersections of bacterial genera between these maternal samples and the first three fecal samples of infant A. Of the 24 genera detected in the infant’s initial microbiome, 13 (54 %) were also present in the maternal microbiome: 10 shared with maternal stool and 3 with the oral microbiome. (Fig. S3, Table S4). Across all infants, the number of genera detected in the infant stool microbiome increased throughout the first year (Fig. S4), with the composition gradually resembling the maternal fecal microbiome (Figs. 3A and 4A).

### Effects of early life events on the development of the fecal microbiome

Infant health records and detailed developmental information were collected throughout the year *via* a diary. Factors known to influence the gut microbiome were monitored (Fig. 1; Table S1) and differences in abundances of the 55 most abundant bacterial genera before, during (for longer-lasting events, such as probiotic consumption), and after each event were statistically tested to assess their impact (see Methods). In the frequently sampled infant A, we identified significant effects of major factors such as probiotic consumption, diet (breastfeeding vs. solid foods), and vaccination (Table S5). For the weekly sampled infants B-M, the influence of these factors was pair/tested by pooling individual available samples, taking one sample per infant before, during (if long enough), and after the event (Table S6). The effect of probiotic consumption was tested separately for each infant B-M by pooling all the available samples in the defined periods. Specific results are described below.

### Diet – breastfeeding vs. solid food introduction

The transition from breastfeeding to the addition of solid foods led to gradual microbiome changes (Fig. S5). In infant A, the introduction of solid food (from week 19 onward) triggered significant shifts in most of the detected genera (Fig. 3, Table S5, Fig. S6). Initially, with low diet diversity, few changes (4 out of 55 bacterial genera) were observed, but as diet diversity increased over the following two months, 44 out of 55 bacterial genera exhibited statistically significant changes. These shifts were linked to the emergence of "Later-colonizers". Some of the changes were directly related to specific foods, for example, *Turicibacter* appeared four days after the first potatoes were introduced, and *Lactococcus* appeared almost directly after the first consumption of legumes. Additionally, higher consumption of plant polysaccharides significantly increased the abundance of *Lachnospiraceae* (*p* < 0.05). In the weekly sampled infants (B-K and M), fluctuating bacterial species abundance was observed, but most results were not statistically significant. Similarly, we were unable to identify direct changes associated with specific foods (Table S6, Fig. S4).

### Effect of colonization with probiotic strains

Infants who received probiotic preparations of *Escherichia, Lactobacillus,* and *Bifidobacterium* were included in this analysis. Importantly, the decision to administer probiotics was made independently by the infants' parents or pediatricians and was not part of our study. Infants A and E received the probiotic Colinfant New Born (Dyntec; Czech Republic), which contains *Escherichia coli* strain A0 34/86. The successful colonization of *E. coli* in both infants during the first year of life has been previously reported (Micenková et al., 2020, 2024).

**Fig. 6:**
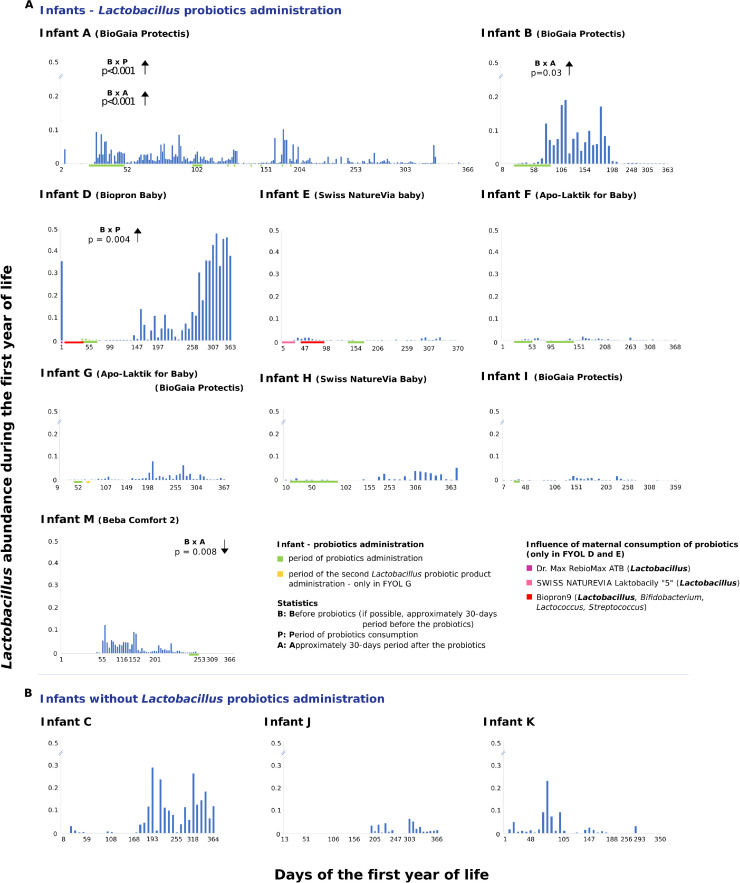
**The effect of*****Lactobacillus*****probiotic administration on*****Lactobacillus*****abundance during the first year of life. A.** Infants administered various *Lactobacillus* probiotics (or a combination of *Lactobacillus* with *Bifidobacterium*). Comparison of abundance before, during, and after probiotic administration, with statistical significance (Kruskal-Wallis test with Dunn's post hoc). **B.** Control infants without *Lactobacillus* probiotic administration.

Infants A, B, and D-I received probiotics containing *Lactobacillus* during the first three months of life (days 12–60), infant M in days 236–248. In frequently sampled infant A, *Lactobacillus* abundance increased after consuming the probiotic BioGaia (Biogaia; Sweden), which contains *L. reuterii* Protectis® (from 0.0 % before consumption to 9.3 %, on the third day). This genus persisted in the fecal microbiome throughout the year, with a median of 0.5 % and a peak of 10.1 % detected immediately after additional probiotic doses in week 25. In the weekly-sampled infants, a significant increase in *Lactobacillus* abundance during or after probiotic administration was observed in only two of eight infants (B and M) (Fig. 6). Statistical comparisons of bacterial composition before, during, and after probiotic consumption are presented in Fig. S7 and detailed in Table S6.

### Vaccination

In the case of infant A, where we monitored microbiome changes over time, an association was observed between two doses of a combination vaccine during the 16th and 25th weeks of life (hexavalent vaccine against diphtheria, pertussis, tetanus, polio, hepatitis B, and *H. influenzae* B) and changes in the microbiome. The abundance of *Clostridioides* significantly increased following vaccination, rising from 0.0 % to 6.0 % one week after the first dose (*p* < 0.01; FDR = 0.01 for comparison 7 days before and after vaccination). *Clostridioides* then became established in the fecal microbiome, fluctuating irregularly with a median value of 0.2 % by the end of the year. No statistically significant microbial changes after vaccination were found when testing the cohort of infants B-M (Table S6).

### Impact of sampling resolution on microbiome variability and event-driven microbiota shifts

To explore the observation that weekly sampling may not accurately reflect microbiota changes, we analyzed the Shannon index variability across individual weeks in infant A (Fig. S8; Table S3). We found that samples within the same week varied by >1 Shannon index point and most of the weekly coefficients of variation of different alpha diversity indices in the first 23 weeks were higher than 10 % (Table S3). While intra-week alpha diversity variability decreased over the first year, beta diversity variability remained high, as indicated by the coefficients of variation of the relative abundances of the bacterial genera (median = 94.4 %, IQR = 57.4 - 155.1 %, Table S3).

To further assess how this variability may influence downstream interpretation, we performed a downsampling analysis of infant A's quasi-daily dataset by selecting one sample per week to simulate a typical weekly sampling regime. To evaluate the impact of reduced sampling on genus-level detectability, we visualized the results using a combination of heatmaps (Fig. S9) and detection frequency boxplots (Fig. S10). Heatmaps allowed for direct comparison of bacterial relative abundance trajectories between the full daily dataset and each weekly subsample, highlighting genera that were missed or appeared only transiently in sparse sampling.

Boxplots in Fig. S10 summarize the weekly detection frequencies across the five subsampled datasets as compared to the detection values from the full daily sampling (red points). The most affected genera were predominantly members of the “Later colonizers group”, where taxa such as *UBA1819, Eggerthella, Subdoligranulum, Granulicatella, Citrobacter, Lachnospiraceae UCG-008, Lachnospiraceae uncultured, Lachnoclostridium 5*, and *Roseburia* were frequently detected in fewer than 5 - and often fewer than 3 - weeks. These low counts make it difficult to distinguish genuine biological presence from random noise (Fig. S9). Among the “Re-appearing colonizers”, sparse sampling often failed to capture their initial transient presence in early life. In several cases - including *Catenibacterium, Senegalimassilia, Dysgonomonas, Faecalibacterium*, and *Alistipes* - this resulted in an apparent shift in their colonization profile, making them resemble “Later colonizers” rather than transient early-life taxa. In other cases, their early presence was detected only sporadically, further complicating efforts to distinguish true biological signals from sampling artifacts.

**Fig. 7:**
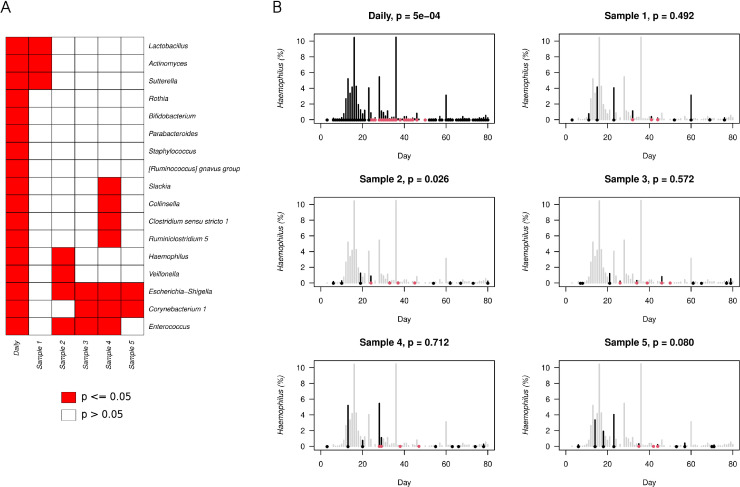
**Effect of subsampling on the detection of probiotic-associated microbial changes: case of*****Haemophilus*****(Infant A). A.** Heatmap of Kruskal–Wallis p-values for bacterial genera affected by probiotic supplementation in Infant A, based on full daily data and five independent weekly subsampling runs. Genera are shown on the y-axis; columns represent sampling schemes. Red indicates statistically significant differences (*p* ≤ 0.05); white indicates non-significant results. **B.** Relative abundance trajectory of *Haemophilus* across the full sampling period. The top left panel shows results from the full daily-resolution dataset; the remaining panels represent weekly subsampling iterations. Grey bars indicate unselected days; black bars mark selected days. Points denote samples used in statistical testing: red points denote samples from the probiotic consumption window; black points denote samples from the 30-day windows before and after supplementation. The y-axis relative abundance (%) of the genus, and the x-axis corresponds to infant age in days. P-values from the Kruskal–Wallis test are displayed in each panel, illustrating the impact of sampling density on detectability of probiotic-associated responses.

Finally, we evaluated the impact of sampling frequency on the detection of microbiota shifts following early-life events. Specifically, two events were examined: administration of probiotics and the first dose of vaccination. In the case of vaccination, *Clostridium* sensu stricto 1 showed a consistent and statistically significant increase in abundance following the first dose when assessed using daily sampling. However, this signal was not detected in any of the subsampled datasets (Fig. S11). This likely reflects both the reduced statistical power due to having only three samples per group and the wider temporal distance of the selected samples from the vaccination event in the weekly subsampling. Similarly, for probiotic supplementation, a total of 17 genera were found to be significantly affected when comparing microbial composition during the 30-day periods before, during, and after administration, based on the full daily sampling. However, none of these genera were consistently detected as significant across all subsampled datasets (Fig. 7A, Fig. S12). Five genera - *Rothia, Bifidobacterium, Parabacteroides, Staphylococcus* and *[Ruminococcus] gnavus* group - were not significant in any of the five subsampling iterations. An additional nine genera, including *Lactobacillus, Actinomyces, Sutterella, Slackia, Collinsella, Clostridium* sensu stricto 1*, Ruminiclostridium* 5*, Haemophilus* (Fig. 7B) and *Veillonella*, were significant in only one of the five subsampling runs.

## Discussion

The first years of life, a period when an infant's gut microbiota is rapidly developing, are considered a "window of opportunity" for microbial modulation (Yang et al., 2019; Gritz and Bhandari, 2015; Laursen et al., 2017). Unlike previous studies (Rodríguez et al., 2015; Bokulich et al., 2016; Nuriel-Ohayon et al., 2016; Zhong et al., 2019; Stanislawski et al., 2018; Palmer et al., 2007; Bergström et al., 2014; Putignani et al., 2014; Nagpal and Yamashiro, 2018; ; ; Wampach et al., 2017;Turroni et al., 2020; Vandenplas et al., 2020; Suárez-Martínez et al., 2023), our study is the first to describe the dynamic nature of the developing intestinal microbiome during the first year and how various early life events shape it. The near-daily sampling of infant A allowed us to track the microbiota's response and the duration of significant bacterial changes in real time.

Early life experiences have complex, long-lasting effects on the acquisition and succession of microbiota during the first years of life. Alterations in gut microbiota and low bacterial diversity are linked to various pathological conditions, including inflammation, atopic eczema, obesity, asthma, and allergies in childhood and adulthood (Francino, 2014; Milani et al., 2017; Pascal et al., 2018; Tanaka and Nakayama, 2017; Lee et al., 2022; Hoskinson et al., 2023). In our study, the near-daily sampling of infant A revealed a gradual increase in bacterial diversity over time alongside longer periods of homogeneous microbial diversity, illustrating the microbiome's progression towards a more stable state*.* Previously published studies have also found that microbiota variability is higher during the first year than in the second and third years of life, reflecting a common pattern in ecological successional systems (Hoskinson et al., 2023; Guittar et al., 2019). Due to the small sample size, we did not assess the effect of gender and instead focused on individual differences.

During the first colonization stage (the first two weeks of life), two groups of bacteria, the "Early-life colonizers" and "Re-appearing colonizers," were established. The "Early-life colonizers" appeared quickly and persisted throughout the year, while the "Re-appearing colonizers" were detected immediately after birth, then were not detected, only to re-emerge later alongside the "Later-colonizers" in the final colonization stage, the "Late-infant" phase. This stage was associated with the introduction of solid foods, medications, probiotic consumption, vaccinations, and other factors.

The most critical influencers during the first weeks of life were colonization with the mother's microbiota (as observed in infant A), diet and probiotic consumption. These factors had immediate effects that lasted throughout the year. While the dense sampling of infant A provides unique insight into the temporal dynamics of early gut colonization, the generalizability of the findings may be limited by the child's strict vegetarian diet and maternal dietary habits. This case should therefore be interpreted as a detailed methodological benchmark rather than a representative model of the general population.

Only 54 % (13/24) of genera in infant A's pioneer microbiome were shared with the maternal microbiome, supporting the view that maternal transmission provides a selective “starter kit“ - with ecological filtering determining which taxa can establish in the neonatal gut. For example, Korpela et al. (2018) showed that infants inherit only specific maternal bacterial classes - primarily Actinobacteria and Bacteroidia - while many maternal taxa (e.g., *Clostridia*) often fail to colonize the infant gut due to ecological filtering and the unique infant gut environment (Korpela et al., 2018). However, in our study, we observed that *Clostridia* were indeed initially transferred from mother to infant but their abundance likely decreased or transiently disappeared probably due to exclusive breastfeeding. Notably, their relative abundance began to increase again after the introduction of complementary foods, suggesting that the changing gut environment supports the re-establishment and growth of these bacteria. Consistent with previous studies (Bokulich et al., 2016; Mortensen et al., 2021), the high abundance of *Lactobacillus* in the mother's vaginal microbiome did not result in higher rates of vertical transfer to the infant's gut, underscoring the limited role of vaginal microbial transfer. Similarly, like in the previously published studies, the proportion of two dominant colonizers, *Bacteroides* and *Bifidobacterium,* during the first week was influenced by breastfeeding. *Bifidobacterium* and *Bacteroides,* enriched in breastfed infants, have metabolic interactions that depend on carbon source type availability. In the absence of *Bifidobacterium, Bacteroides* tends to dominate in the infant gut (Rios-Covian et al., 2013; Lewis and Mills, 2017). A recently published study in which infants were sampled 7 times during the first year of life (at 3, 6, 13, 26, 39, 52, and 78 weeks) found that *Bifidobacterium* and *Bacteroides* appear to be the key organisms that drive microbiota development and consistently predict positive health outcomes (Hickman et al., 2024).

In our study (infant A), *Bacteroides* prevailed 48 h after delivery, when infant formula was the primary diet (before the lactation stabilized). By the 5th day, following the transition to full-breastfeeding, *Bifidobacterium* became dominant. Additionally, the "Re-appearing colonizers" group was transmitted from the mother. This group, transmitted during delivery, may lack traits that facilitate early colonization and instead possess features suited to the mother's adult microbiome. A species will persist in the gut microbiome only if it can access enough resources to reproduce (Guittar et al., 2019). The early-life intestinal environment, especially with a breastfeeding diet, may not provide suitable conditions for the "Re-appearing colonizers". In the case of weekly sampled infants B-K and M, the identification of the "Re-appearing colonizers" was not possible in an unsupervised manner; however, using clustering information from infant A, similar colonization patterns and groups as for infant A were observed. This emphasizes the need for frequent sampling during the first weeks of life, especially for this group of bacteria.

During the breastfeeding period, the infant's less diverse intestinal niche provided ideal conditions for the colonization of probiotic strains. Probiotics containing *Lactobacillus, Bifidobacterium,* and *Escherichia* were administered. When *Lactobacillus* probiotics were given before random colonization began, this genus was immediately detected and became stably established. In infants without probiotic supplementation, stable colonization with randomly acquired *Lactobacillus* and *Bifidobacterium occurred* naturally during the first year. However, the benefits of administering probiotics containing *Bifidobacterium* or *Lactobacillus* to healthy children remain debated (Skórka et al., 2017; Quin et al., 2018). Only limited beneficial clinical effects of probiotics have been described, and there is no reliable evidence to recommend their routine use. The high colonization capacity of the probiotic strain *E. coli* A0 34/86 along with its impact on significant rearrangements in the *Escherichia* population during the first year of life, has already been documented (Micenková et al., 2020, 2024).

Immunization and antigenic stimulation can affect mucosal interactions, regulation, and microbiota development (Skórka et al., 2017). In infant A, immunization with two doses of hexavalent vaccine was associated with an increase in the genus *Clostridioides*. Additionally, an increase in *Flavonifractor* (a genus within the *Clostridia* cluster IV) was observed. While some members of this group have been reported to possess anti-inflammatory properties (Rousseau et al., 2011), our 16S rRNA gene sequencing approach (based on genus-level resolution) does not allow us to determine species-level identity or infer specific functional roles. Moreover, although *Clostridioides* includes *C. difficile*, which is frequently detected in early infancy regardless of toxigenicity (Rousseau et al., 2011), our data cannot distinguish between toxigenic and nontoxigenic strains. It is important to note that the presence of *C. difficile* has been associated with shifts in overall microbial community structure in previous studies (Rousseau et al., 2011; Li et al., 2014); however, in our study, vaccination was not linked to overall changes in bacterial diversity. We acknowledge the limitations of genus-level data in drawing definitive conclusions about biological function or strain-specific effects. No significant post-vaccination changes were observed in infants B–M, which is likely due to high interindividual variability and less frequent sampling. In contrast, the daily sampling in infant A may have increased our sensitivity to detect even subtle shifts.

Diet significantly influences the gut microbiome by providing substrates that promote the growth of various bacteria (Moore and Townsend, 2019). The introduction of solid food marks the second key window of development for the intestine and immune system (Houghteling and Walker, 2015). In our study, solid food was introduced to infant A at 5 months, alongside continued breastfeeding. The addition of different foods impacted microbiome resilience and increased alpha diversity, as reflected by a higher Shannon diversity index. A primarily plant-based diet was associated with a re-appearance of genera such as *Lachnospira, Collinsella, Slackia, Blautia*, and *Dorea -* early colonizers related to the adult microbiome, which had been detected after delivery and then disappeared. With the introduction of solid food, the variety of available nutrients expanded, creating new ecological niches for different bacteria (Vallès et al., 2014). Association between various foods and the first appearance of certain bacteria was observed (e.g., *Turicibacter* with potatoes, *Lactococcus* with legumes). Previous studies using 16S rRNA gene sequencing have reported that resistant starches from potatoes are associated with an increased abundance of the genus *Turicibacter* (Bibi et al., 2019). Similarly, in our study, the introduction of potatoes into the infant diet coincided with the first detection of *Turicibacter* in the gut microbiota. While mechanistic or functional interpretations cannot be drawn from genus-level data, this observation points to a potential association between early dietary exposure to resistant starches and the presence of *Turicibacter* during gut microbiota development. The abundance of *Lachnospiraceae* was positively associated with increased consumption of plant polysaccharides, suggesting that dietary intake of these complex carbohydrates may promote the proliferation of this bacterial family. *Lachnospiraceae* are known to be primary degraders of plant biomass, utilizing their enzymatic capabilities to break down polysaccharides into simpler compounds (Zaplana et al., 2024). In line with our observations, Vatanen et al. reported similar patterns at the level of microbial metabolites, noting a gradual decline in enzymes characteristic of *Bifidobacterium*, such as l-lactate dehydrogenase, coinciding with the cessation of breastfeeding. Concurrently, there was an increase in enzymes like transketolase, associated with fiber metabolism, reflecting the introduction of solid foods. These functional shifts mirror the transformation of the early infant gut microbiome into a more adult-like community, increasingly adapted to diverse and fermentative dietary substrates (Vatanen et al., 2018). In our study, in infant A, the use of high-frequency sampling enabled detection of the initial presence of the so-called "Re-appearing colonizers" shortly after birth - an observation likely missed with less frequent sampling - and allowed us to distinguish them clearly from the "Later colonizers" that emerged during the dietary diversification phase. Our findings contribute to the growing body of research on gut microbiome succession and maturation in infancy by offering high-resolution temporal insights that complement large-scale metagenomic studies. Similar to Bottino et al. (2025), who used gut microbial taxonomic relative abundances from metagenomes to estimate microbiome age, we observed consistent patterns of decline in *Bifidobacterium* and an increase in *Faecalibacterium* with advancing infant age. Notably, *Faecalibacterium* appeared in our study as a "later colonizer," emerging during the final colonization phase associated with dietary diversification and cessation of exclusive breastfeeding (Fahur Bottino et al., 2025).

Assessing the impact of diet is challenging because it involves long periods of time during which not only diet changes, but also growth, activity, and interactions with the environment. It is difficult to attribute this development solely to diet, so it would be ideal if future studies compared infants exclusively breastfed for longer periods with infants who switched to a solid food earlier.

While simethicone use in infant A is unlikely to have influenced the microbiome composition directly, its use reflects the individual variation in early infant gut physiology. Of note, a second infant from the same mother, also raised on a strict vegetarian diet, did not require similar interventions, suggesting that simethicone use was not systematically linked to maternal diet or behavior.

The presence of older siblings in some households may have contributed to increased microbial exposure, as has been suggested in other studies. While our sample size did not allow for a formal analysis of this variable, it remains a relevant environmental factor for future microbiome research in early childhood.

While infant A exhibited robust and statistically significant genus-level microbiome shifts following the introduction of solid foods, these events were not detectable - at a cohort level - in infants B–M. The weekly sampling framework provided limited pre- and post-introduction data points per infant and captured a wide range of complementary foods, reducing the power to detect consistent dietary effects across individuals. However, the introduction to solids was associated with the appearance of the group of "Re-appearing colonizers" and "Later-colonizers". In the less frequently sampled infants, most had only two high microbial diversity (HMD) periods, with the latter often coinciding with greater dietary diversity. This pattern supports the notion that increased food variety contributes to enhanced microbiome complexity, even if not statistically significant in sparse datasets (Homann et al., 2021). Ecological succession of gut microbiota has been a central theme of gut ecology for over 120 years. Despite the significant impact on life-long health, the process of bacterial succession during infancy remains poorly understood. The intestinal microbiome responds to and is shaped by environmental factors - "everything is everywhere, but the environment selects" (Xiao et al., 2021). Bacterial metabolites, which reflect the functional activity of the whole microbiota, can be used as additional non-invasive markers to assess the functional status and development of the gut microbiota in humans from the first days of life (Aust et al., 2024).

To evaluate the impact of sampling resolution on microbial community analysis, we performed a downsampling analysis on the near-daily time series of infant A. Five independent weekly subsampling runs were created and analyzed using the same statistical methods applied to the full dataset. The results showed a substantial reduction in sensitivity to detect event-related microbiota shifts. For example, while *Clostridium* sensu stricto 1 consistently increased following the first dose of vaccination in the full daily dataset, this signal was not detected in any of the subsampled versions. The limited temporal resolution (three samples before and after the event spanning three weeks) likely failed to capture the narrow window of microbiome perturbation. Similarly, only a minority of the 17 genera that responded significantly to probiotic consumption in the full dataset remained detectable in one or more subsampled versions, with no genus retained across all iterations. These findings further show how low temporal resolution can obscure transient but biologically meaningful microbial shifts. Beyond event-driven responses, our downsampling analysis revealed broader implications for the ecological interpretation of colonization patterns. Heatmaps and detection frequency boxplots highlighted that sparse sampling frequently failed to capture the initial appearance or reappearance of certain genera - particularly those categorized as "later" or "reappearing colonizers." In several cases, these genera were nearly absent or entirely missed across weekly subsampled series, potentially leading to misclassification or underestimation of their role in microbial succession. Genera such as *Faecalibacterium, Alistipes*, or *Subdoligranulum*, which exhibited dynamic and intermittent trajectories in the daily data, often appeared only sporadically or not at all in downsampled versions. These omissions are not merely technical artifacts - they carry significant consequences for how we interpret early-life colonization dynamics and the developmental trajectories of the infant gut microbiome. Our approach builds on previous work of (Koenig et al., 2011), who demonstrated the value of longitudinal infant microbiome sampling by identifying co-occurrence patterns and ecological transitions in a single infant followed over 2.5 years. While that pioneering study provided crucial early insights, the sampling frequency - approximately 60 time points - was considerably lower than in our near-daily dataset. By increasing the temporal resolution within the critical first year of life, we were able to observe highly dynamic colonization patterns, such as short-lived "Re-appearing colonizers," which are unlikely to be detected with sparser sampling.

While our study offers a detailed characterization of early gut microbiome development through high-frequency sampling in one infant, the small cohort size and sparse sampling in the second cohort limited the application of more complex statistical frameworks, such as age-adjusted mixed-effects models. We acknowledge this as a methodological limitation. Although the near-daily dataset was available for only one infant (*n* = 1) and the broader weekly cohort comprised 12 infants (*n* = 12), our analytical design accounted for this imbalance. We refrained from making cohort-level generalizations unless statistically supported and interpreted all findings from infant A as exploratory. However, our study was not designed to substitute for population-level inference but rather to expose the constraints of low-resolution designs. At the same time, this challenge highlights an important insight: capturing the nuanced, event-driven dynamics of microbiota development requires denser longitudinal sampling and larger cohorts. Our findings thus underscore the need for future studies designed with sufficient resolution to support robust statistical modeling of early microbiome trajectories. Based on our experience, we recommend that future studies aiming to evaluate the effects of discrete early-life events - such as vaccination, dietary transitions, or probiotic administration - include dense sampling around the event of interest in at least a small subset of participants. This approach can guide the selection of appropriate timepoints and sampling frequency, ensuring that transient but biologically relevant microbial changes are not missed.

## Conclusions

In our study, we provided the first detailed description of bacterial succession during the first year of life, highlighting the dynamic nature of the developing gut. While similar patterns of bacterial colonization and stages were observed in both near - daily and weekly sampling, our initial hypothesis - that weekly sampling might miss key nuances - was supported. Less frequent weekly sampling limited our ability to capture immediate microbiome changes in response to specific events such as diet change, probiotics consumption, and vaccination. This underscores the importance of conducting pilot studies with daily sampling around critical events to determine the optimal sampling frequency and sample size for capturing meaningful microbial shifts. Incorporating such high-resolution windows, even in a small subgroup of participants, may improve the detection of transient yet biologically meaningful microbial changes.

Supplementary material

**Fig. S1. Study design of the project FYOL (First Year Of Life).**

**Fig. S2. Principal component analysis of gut microbiota composition across the study cohort. A.** Infant A based on near-daily sampling. Points are colored by age in days. **B.** Infants B-K and M; colors represent age in weeks. **C.** Infants B–K and M, with color lines representing individual subjects.

**Fig. S3. The UpSet diagram distinguishes unique and shared infant A and mother A microbiomes.** Amplicon sequence variants (ASVs) were filtered to ensure robust detection. In maternal samples collected around the time of delivery, only ASVs with ≥3 reads per sample were considered present. For the first three infant stool samples, an ASV was considered present if it had ≥3 reads in at least one of the three samples, or ≥1 read consistently across all three samples. *ASV genera level overlap was then defined as the presence of at least one ASV belonging to a given genus across the compared matrices.

**Fig. S4. Number of bacterial genera detected during the first year of life in infants and mothers in the quasi-daily sampled infant A (top) and infants B-M (bottom).**

**Fig. S5. Number of bacterial genera and Shannon index value observed during the first year of life of infants B-K (top) and M (bottom).**

**Fig. S6. Results of testing of differential bacterial abundance across four time periods following solid food introduction, as compared to breastfeeding in infant A (left) and infants B-M (right).** Only genera with at least one significant change are shown.

**Fig. S7 Results of testing of differential abundance of bacterial genera during (left) and after (right) probiotic intervention compared to baseline.** Only genera with at least one significant change are shown.

**Fig. S8. Shannon diversity index across individual weeks during the first year of life.** Each box represents variability within one calendar week.

**Fig. S9. Comparison of genus-level abundance across full daily data and weekly subsampling (Infant A).** Heatmaps show the relative abundance of bacterial genera over time in Infant A for the full daily dataset (“Daily”) and five independent weekly subsampling runs. Each row represents one genus, and each column represents a sampling day. Genera are grouped by colonization category: “Early-life colonizers”, “Re-appearing colonizers”, and “Later colonizers”. While dominant early colonizers are consistently detected across all datasets, many reappearing and later colonizers are inconsistently captured or appear entirely absent in the downsampled runs. White indicates missing data.

**Fig. S10. Detection frequency of bacterial genera in weekly subsampling compared to full daily data (Infant A).** Boxplots show the distribution of weekly detection frequencies across five random subsampling runs (*n* = 52 weeks each) for each genus. Red triangles represent weekly detection frequencies from the full, high-resolution daily sampling (“Daily”), indicating the maximum number of weeks each genus was detected in Infant A. Genera are grouped by colonization type — “Early-life colonizers” (blue), “Re-appearing colonizers” (green), and “Later colonizers” (gray) — and ordered within each category by decreasing detection in the daily sampling. Dotted lines at 3 and 5 weeks indicate reference thresholds for evaluating detection consistency: 3 weeks reflects a minimal count for observing variability across time, while 5 weeks suggests slightly higher recurrence. These thresholds are not based on prior microbiome studies but reflect general statistical considerations for temporal detection. While early colonizers remain robustly detected even with sparse sampling, later and reappearing colonizers show reduced detectability in weekly subsamples.

**Fig. S11. Impact of subsampling on the detection of *Clostridium* sensu stricto 1 increase following vaccination in Infant A.**

Each panel shows the relative abundance trajectory of *Clostridium* sensu stricto 1 across time. The top left panel displays the full daily-resolution dataset, while the remaining five panels represent five independent weekly subsampling runs. Grey bars indicate days not selected in a given subsample; black bars indicate selected days. In each panel, the red point marks the sample from the day of the first vaccine dose. Black points indicate samples used for statistical testing: in the daily data, these include samples from the 7-day intervals before and after vaccination; in the subsampled datasets, they include three weekly samples before and after the vaccination week. The y-axis shows relative abundance (%), and the x-axis indicates infant age in days. P-values from the Wilcoxon rank-sum test (CLR-transformed data) are shown, demonstrating how sparse sampling may obscure transient microbial shifts associated with vaccination.

**Fig. S12. Effect of subsampling on the detection of probiotic-associated microbial changes (Infant A).**

Each panel displays the relative abundance trajectory of a bacterial genus that showed a statistically significant response to probiotic supplementation in the full daily-resolution dataset (Kruskal–Wallis test, CLR-transformed data). The top left panel shows results based on the full daily sampling of Infant A; the five remaining panels represent five independent weekly subsampling runs. Grey bars indicate days not selected for a given subsample; black bars mark selected days. Points represent samples used in statistical testing: red points denote samples from the probiotic consumption window; black points denote samples from the 30-day intervals before and after supplementation. The y-axis shows relative abundances in% for the respective genus, and the x-axis corresponds to infant age in days. The p-value of the Kruskal–Wallis test is displayed in each panel, illustrating how sparse sampling can obscure detection of transient microbial responses.

**Table S1.** Information about the infants (*n* = 13) and mothers and all samples analyzed in this study.

**Table S2.** List of primers and the length of PCR products.

**Table S3.** Coefficients of variation of various alpha diversity indices and relative abundances of different genera measured within each week of the first year of life of infant A.

**Table S4.** Composition of the infant's A early gut microbiome - overlap between the maternal A and infant A microbiomes.

**Table S5.** Results of statistical testing of the influence of early life events on the bacteria composition at the genera level in infant A.

**Table S6.** Results of statistical testing of the influence of early life events on the bacteria composition at the genera level in sparsely sampled infants B-M.

## Ethics approval and consent to participate

TNG study (Central European Longitudinal Study of Parents and Children: The Next Generation) was approved by the Ethics Committees of the University Hospital Brno, the Czech Republic (Ref. No. 20140409-01) and follow up inside the FYOL study by the CELSPAC Ethics Committee, Masaryk University, Brno, the Czech Republic (Ref. No. CELSPAC/EK/1/2018).

## Consent for publication

Not applicable.

## Data availability

The data underlying this article are available in the article and in its online supplementary material. The metagenomic data for this study have been deposited in the European Nucleotide Archive (ENA) at EMBL-EBI under accession numbers PRJEB82126 (infant A) and PRJEB82127 (infants B-M).

## Funding

This project was supported by the European Union's Horizon 2020 research and innovation programme under grant agreement No 857560.

This publication reflects only the author's view and the European Commission is not responsible for any use that may be made of the information it contains.

This work was supported by the project National Institute of Virology and Bacteriology (Programme EXCELES, ID Project LX22NPO5103) - Funded by the European Union - Next Generation EU (author LM).

## Authors' contributions

Methodology: LM, VT, PV, EB. Investigation: LM, JM, KB, DK. Analysis: SS, BL, BZ. Writing and editing: LM, SS, EB. All authors have read and agreed to the published version of the manuscript.

## Declaration of competing interest

The authors declare that they have no competing interests.

## Appendix Supplementary materials

Image, application 1

Image, application 2

Image, application 3

Image, application 4

Image, application 5

Image, image 6

Image, image 7

Image, application 8

Image, application 9

Image, application 10

Image, application 11

Image, application 12

Image, application 13

Image, application 14

Image, application 15

Image, application 16

Image, application 17

Image, application 18
