# Supplementary material for: Sampling frequency matters: mapping of the healthy infants' gut microbiome during the first year of life
Source: Curr Res Microb Sci. 2025 Sep 9;9:100470. doi: 10.1016/j.crmicr.2025.100470 (PMC12466160; doi:10.1016/j.crmicr.2025.100470)
Supplement: Supplementary file 1 [file mmc1.pdf]

PROJECT FYOL - First Year Of Life

SAMPLING

PILOT STUDY - Infant A

12 FYOL infants (B-M)

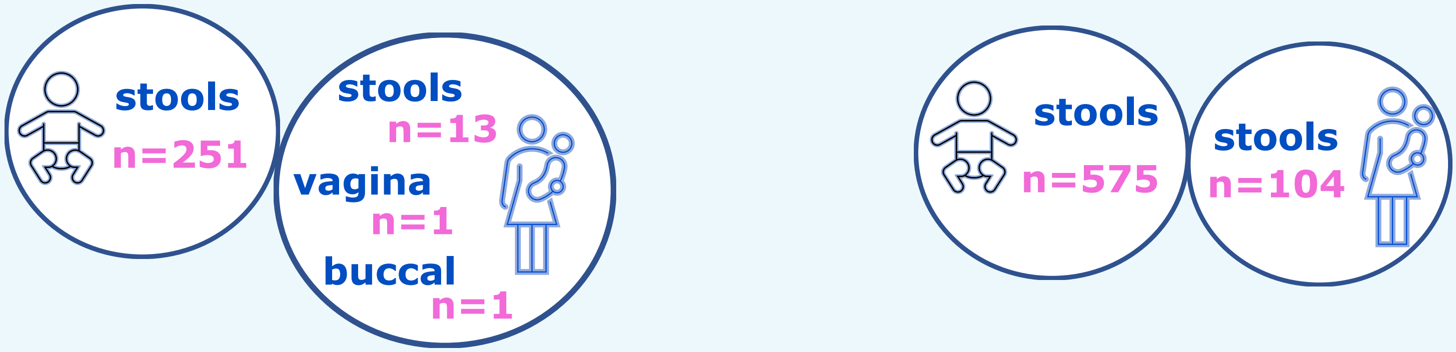

RECORD DIARY

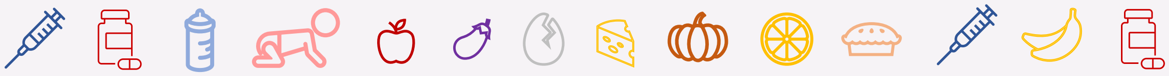

SAMPLING KITS

Flocked swabs (stools from infants, vagina and buccal swabs)

Stool container (stools from mothers)

DNA ISOLATION KIT

DNeasy PowerLyzer PowerSoil kit (QIAGEN)

gDNA QUALITY CHECK

Nanodrop - DNA yield and purity

PCR AMPLIFICATION

V4 region (515F-806R) of bacterial 16S rRNA gene

LIBRARY PREPARATION

16S Metagenomic Sequencing Library Preparation Protocol (Illumina)

SEQUENCING

MiSeq reagent kit v2 - 500 cycles (Illumina)

BIOINFORMATIC ANALYSIS

QIIME 1.9.1

STATISTICAL ANALYSIS

R3.6.1
