# Supplementary figures and images for: Sampling frequency matters: mapping of the healthy infants' gut microbiome during the first year of life
Source: Curr Res Microb Sci. 2025 Sep 9;9:100470. doi: 10.1016/j.crmicr.2025.100470 (PMC12466160; doi:10.1016/j.crmicr.2025.100470)

A

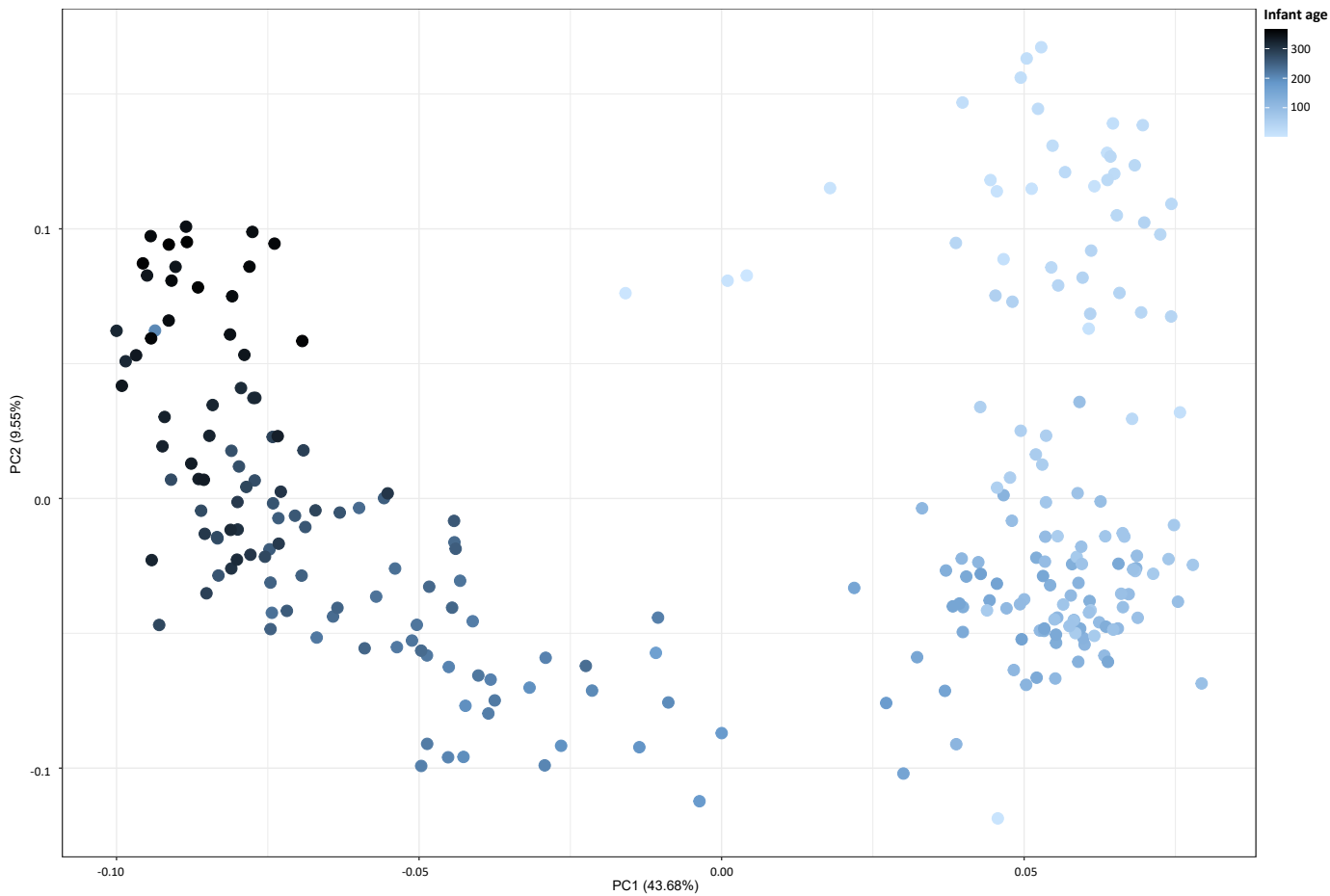

B

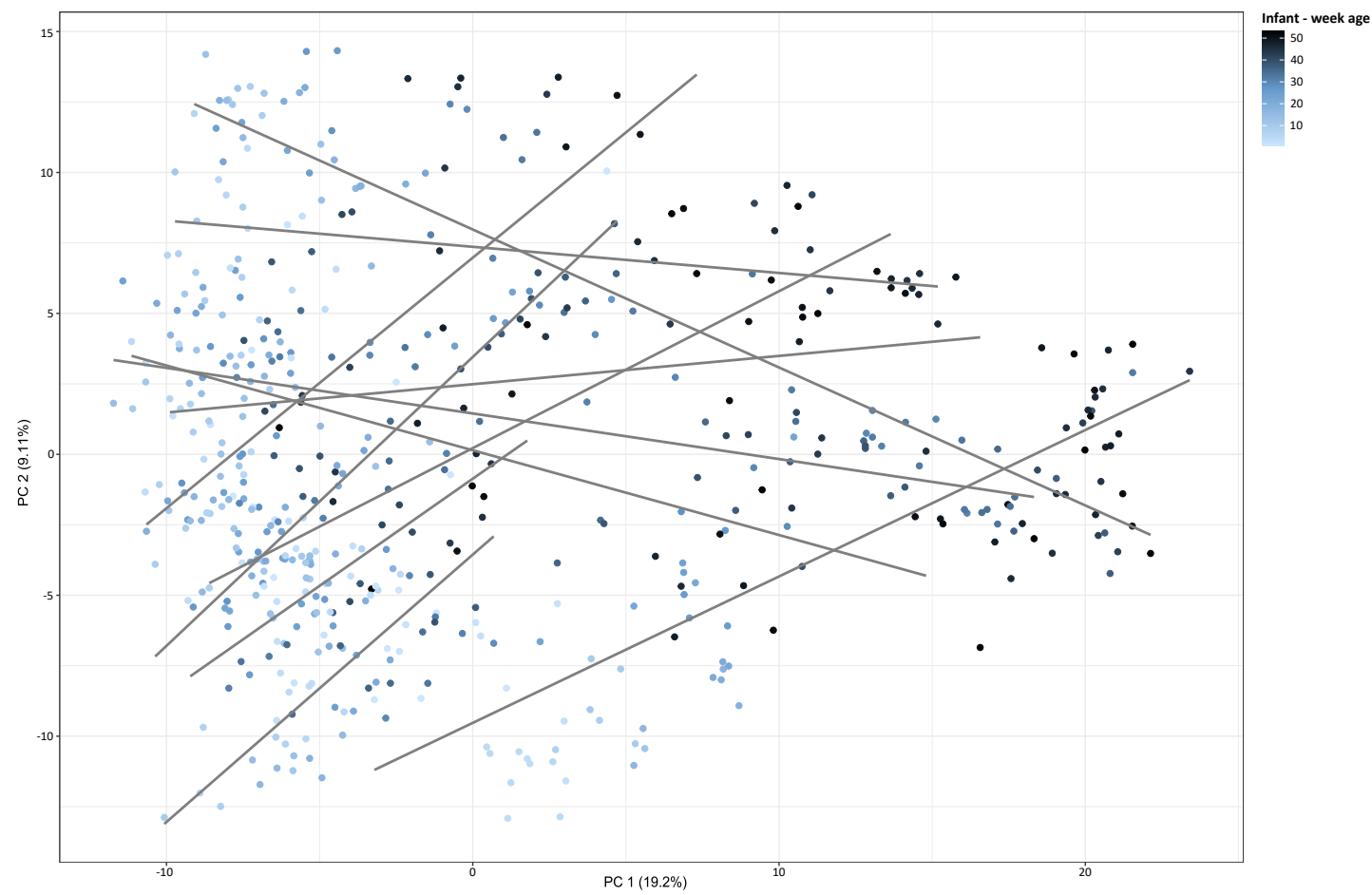

C

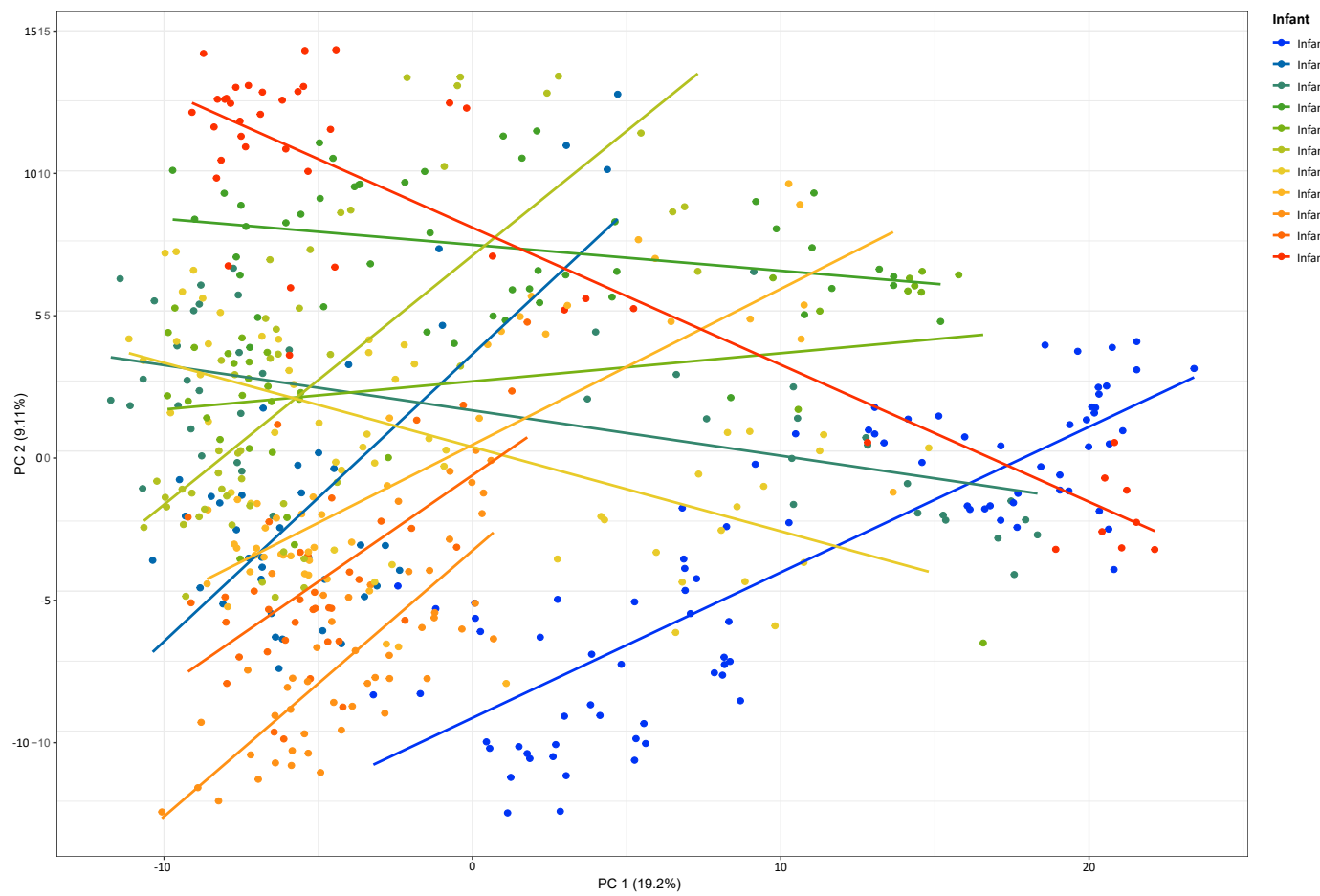

Supplement: Supplementary file 2 [file mmc2.pdf]

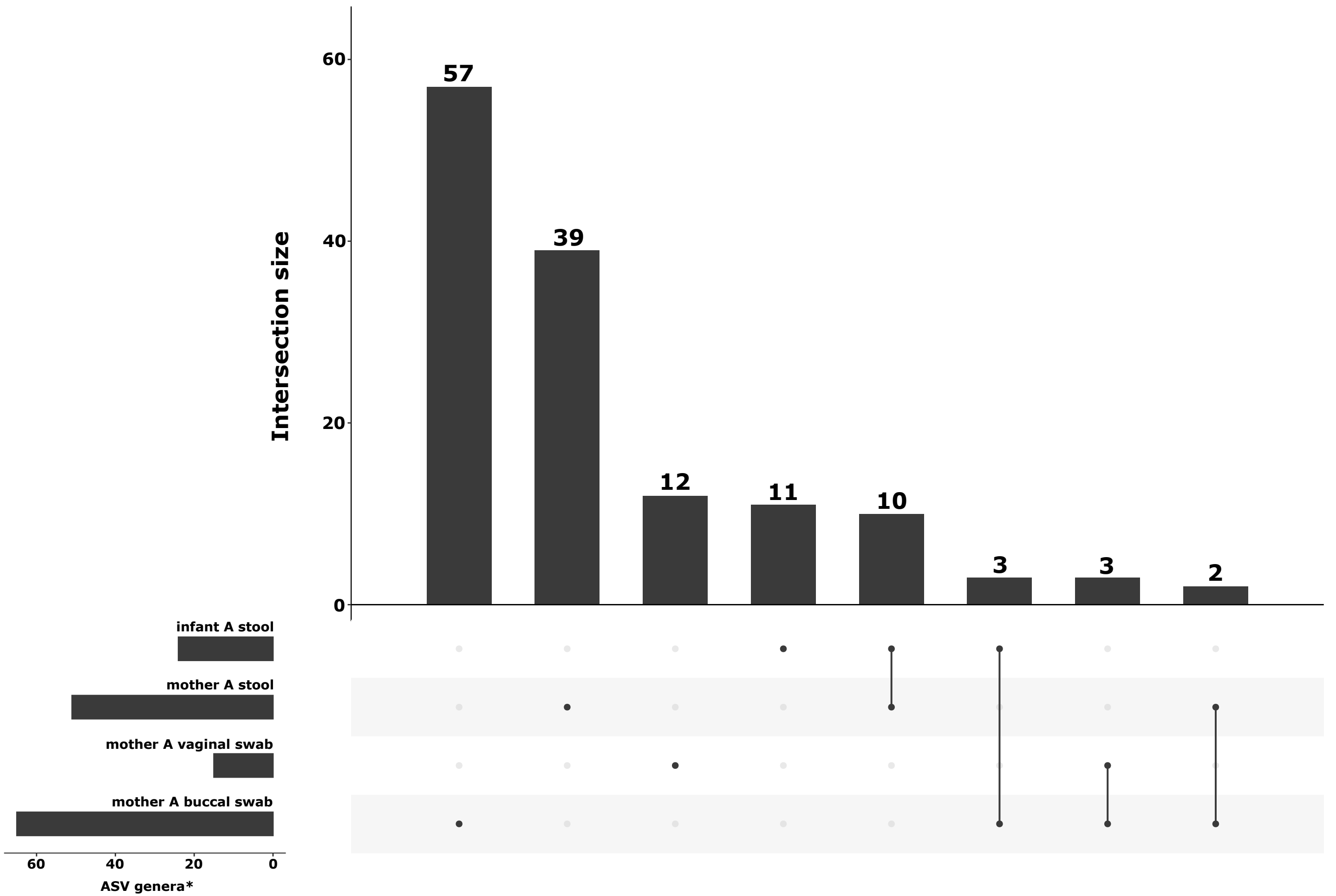

Supplement: Supplementary file 3 [file mmc3.pdf]

Infant A

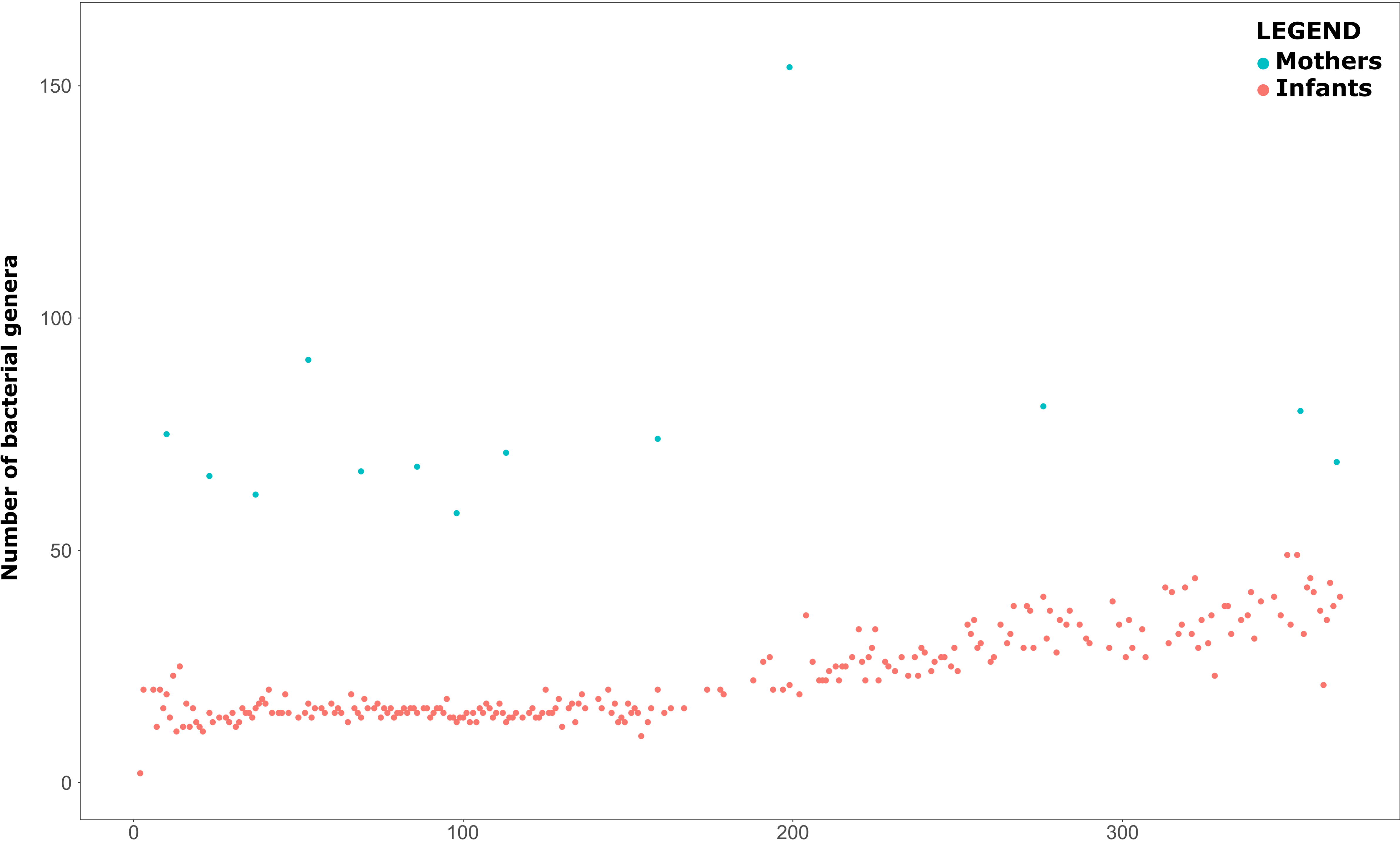

Infants B, C, D, E, F, G, H, I, J, K, M (n = 11)

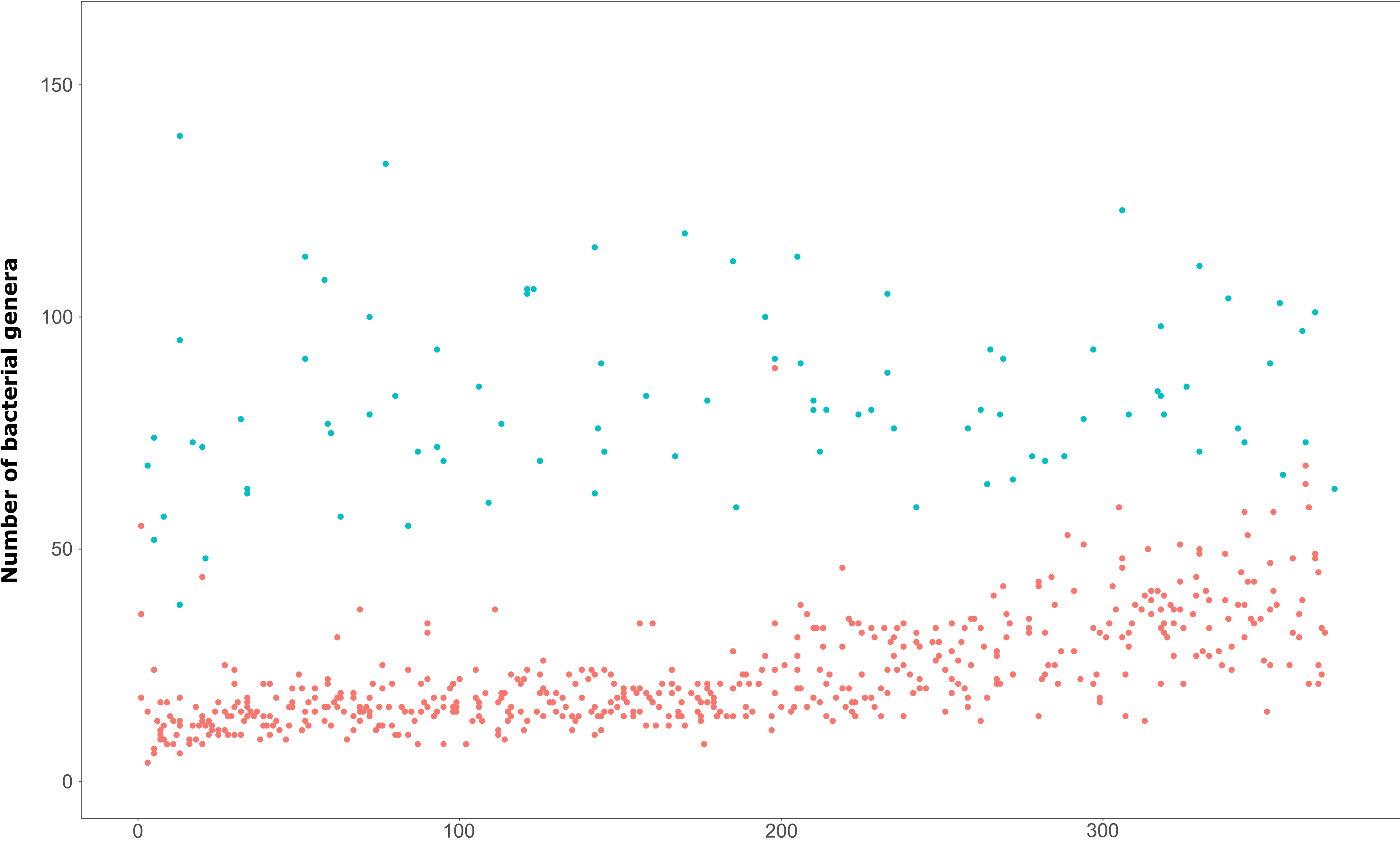

Supplement: Supplementary file 4 [file mmc4.pdf]

Index value

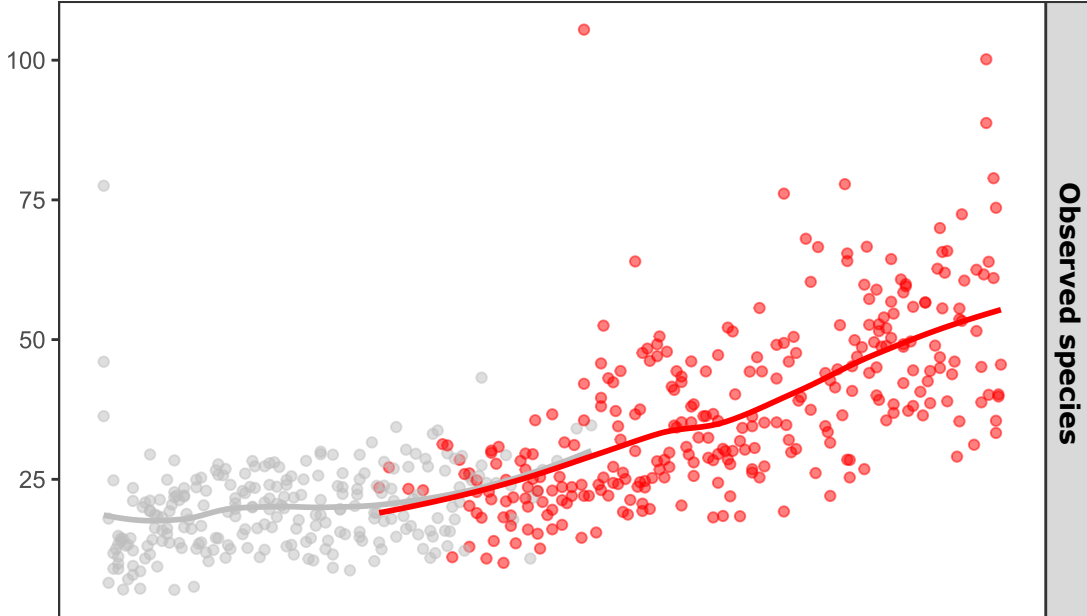

Solid food

0

1

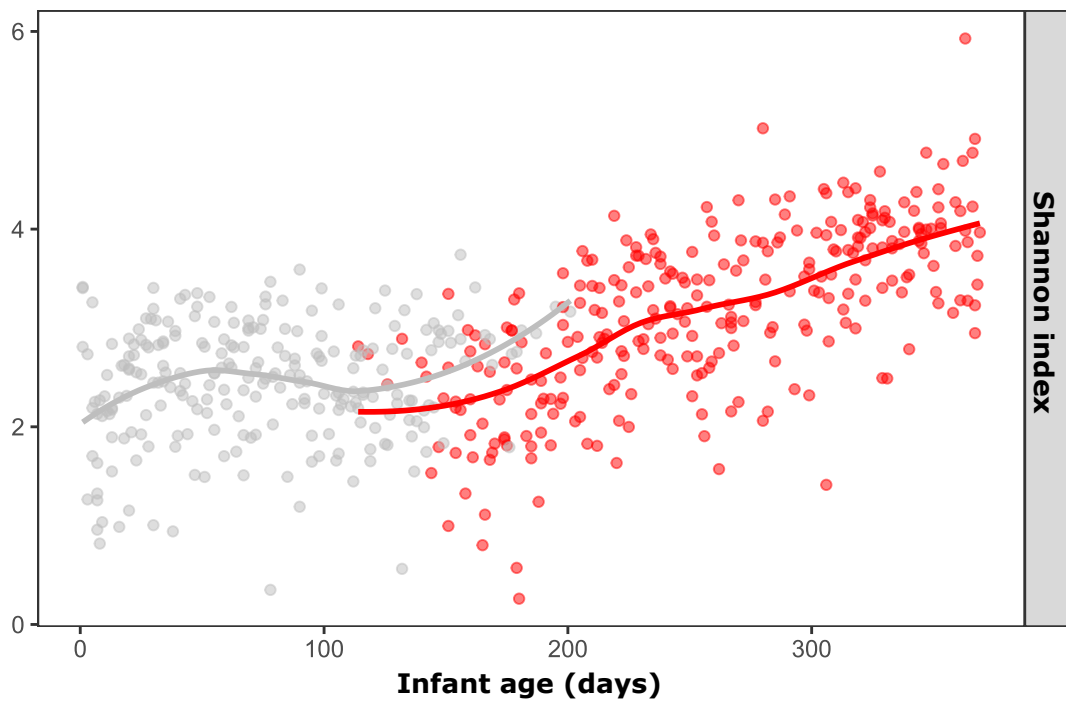

Infant age (days)

Supplement: Supplementary file 5 [file mmc5.pdf]

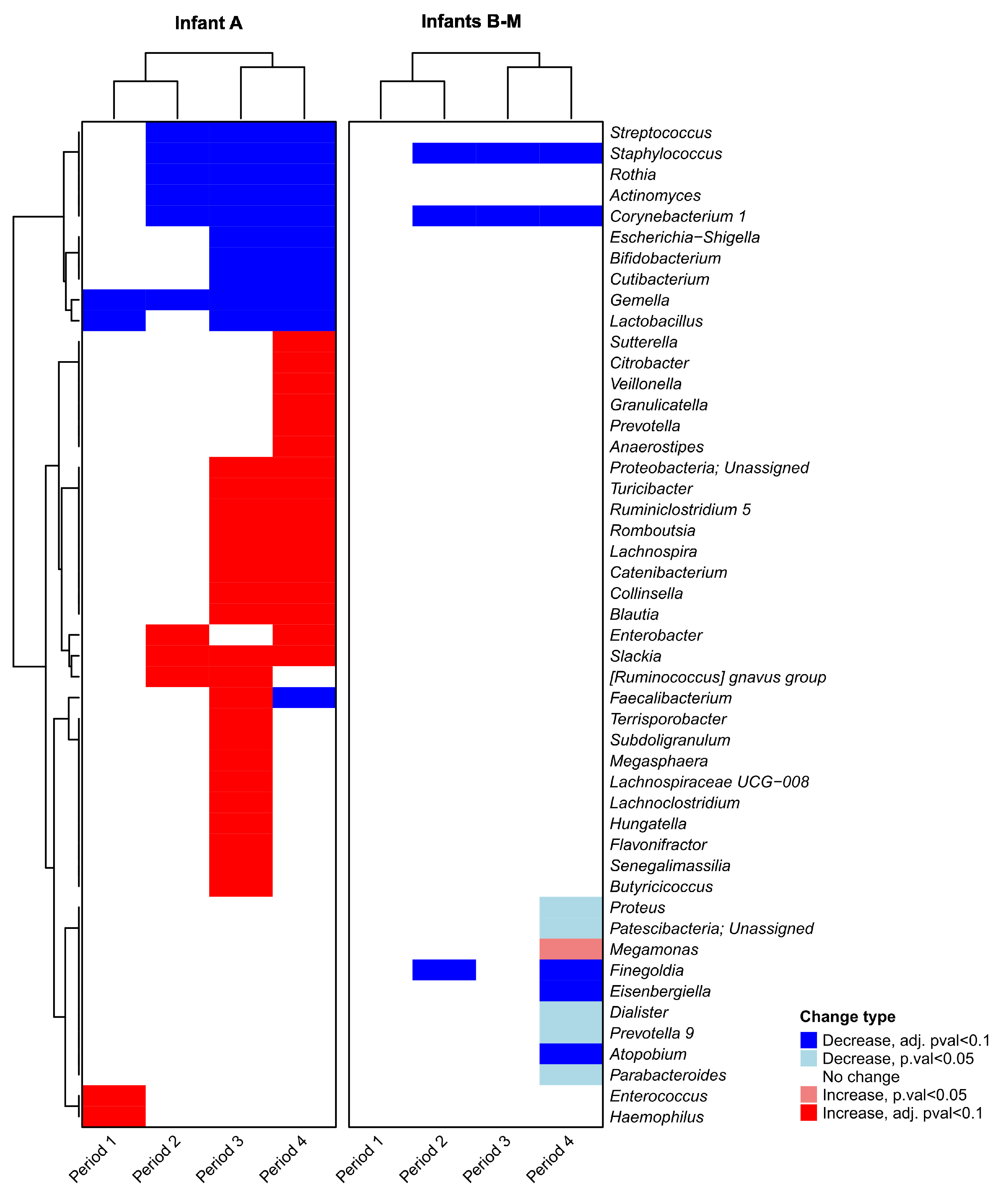

Supplement: Supplementary file 6 [file mmc6.jpg]

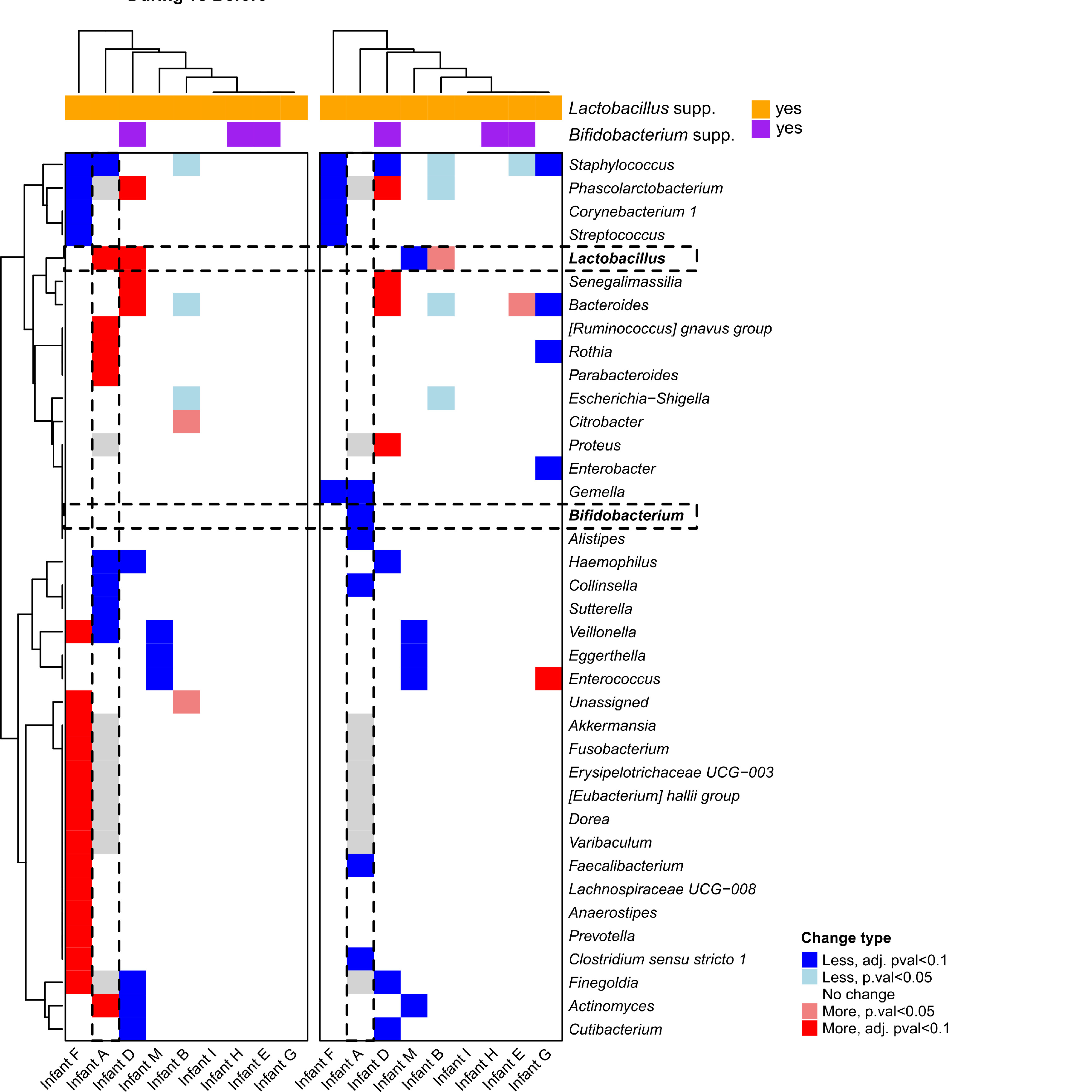

Supplement: Supplementary file 7 [file mmc7.jpg]

Shannon diversity index

Infant age

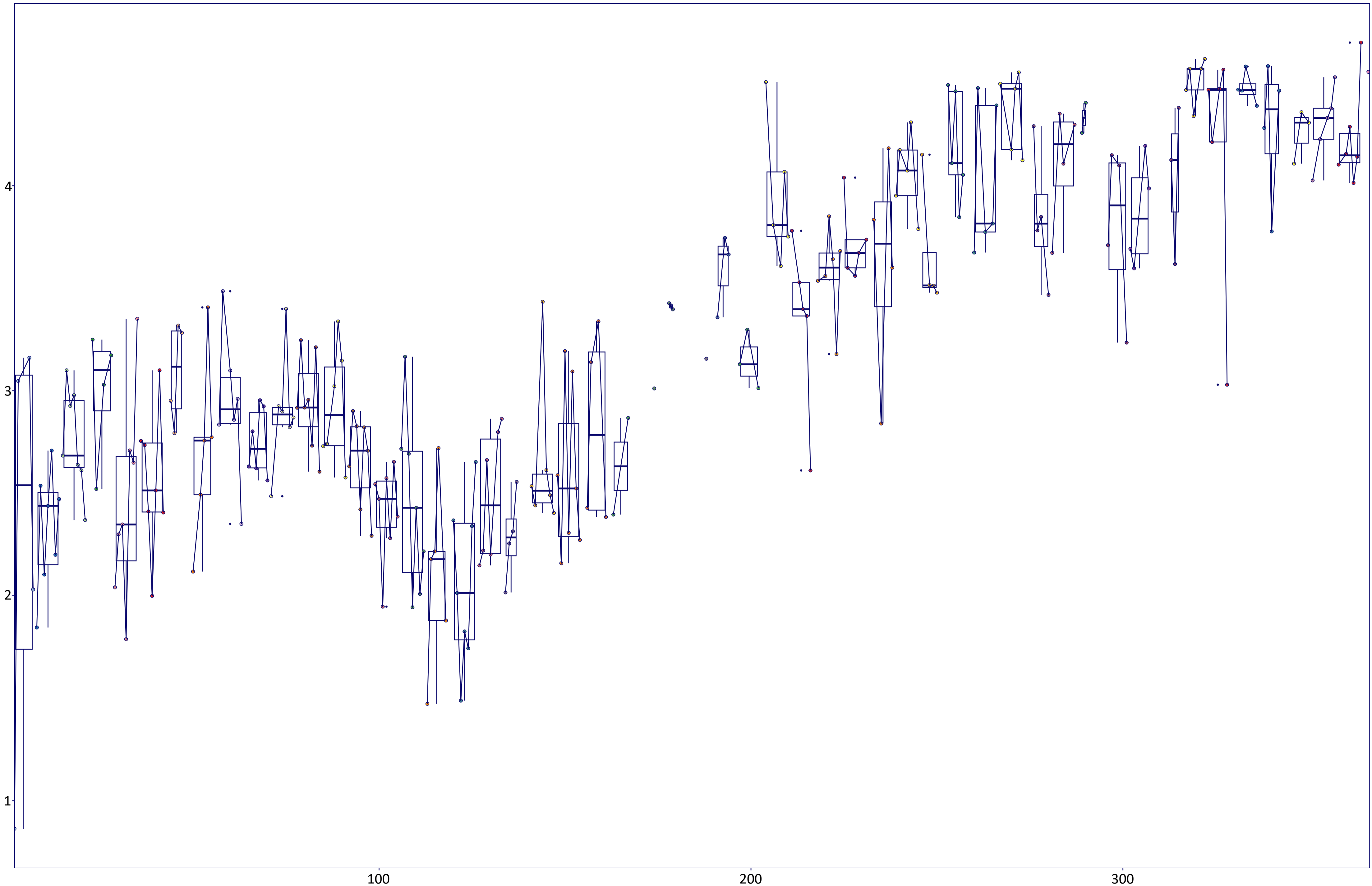

Supplement: Supplementary file 8 [file mmc8.pdf]

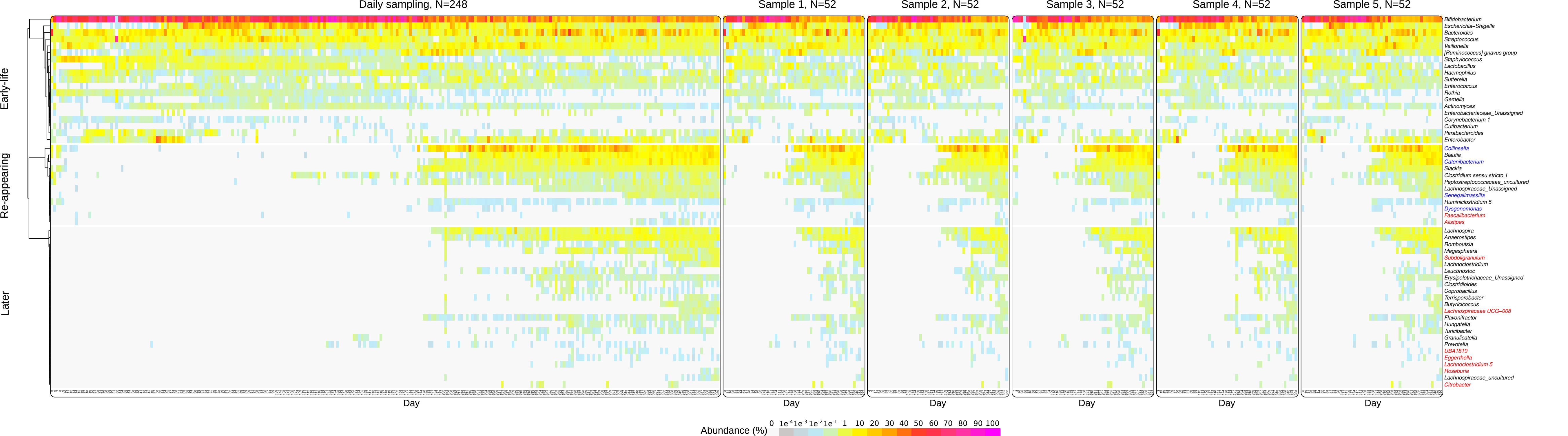

Supplement: Supplementary file 9 [file mmc9.pdf]

# Colonization Group

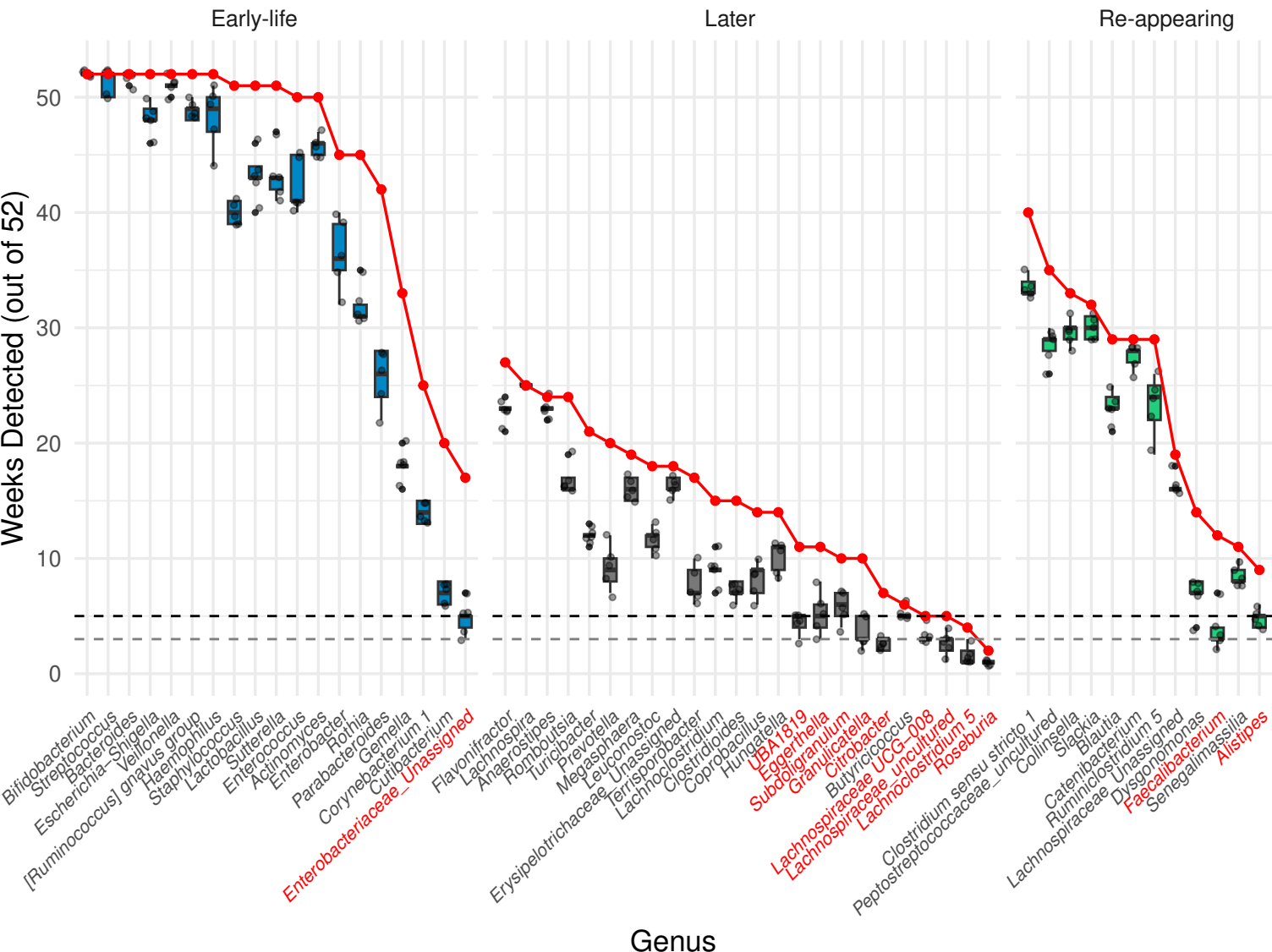

Supplement: Supplementary file 10 [file mmc10.pdf]

**Daily,  $p = 6e-04$**

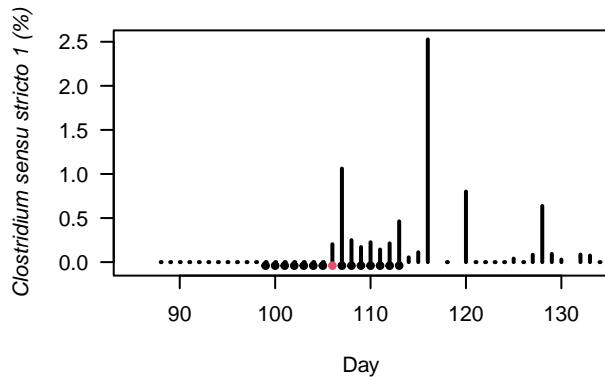

**Sample 1,  $p = 0.100$**

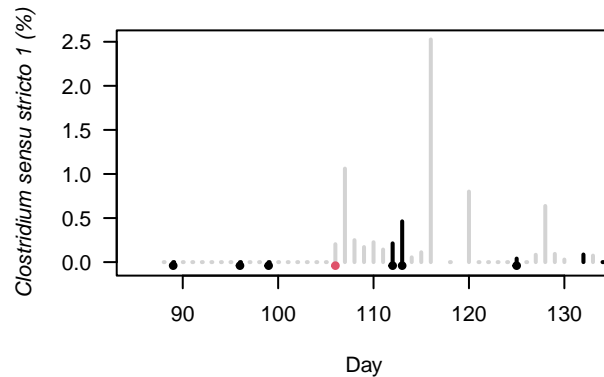

**Sample 2,  $p = 0.100$**

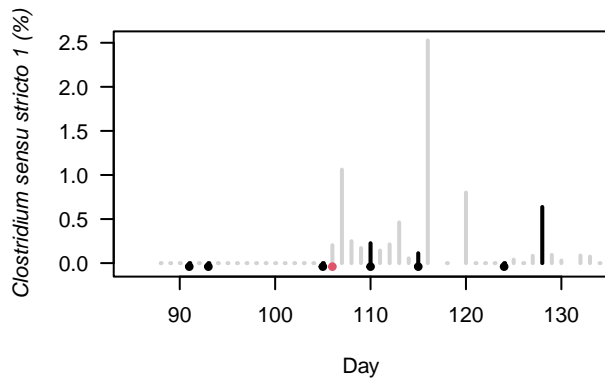

**Sample 3,  $p = 0.200$**

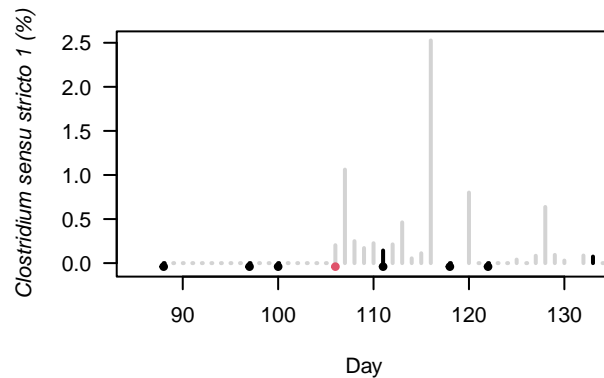

**Sample 4,  $p = 0.100$**

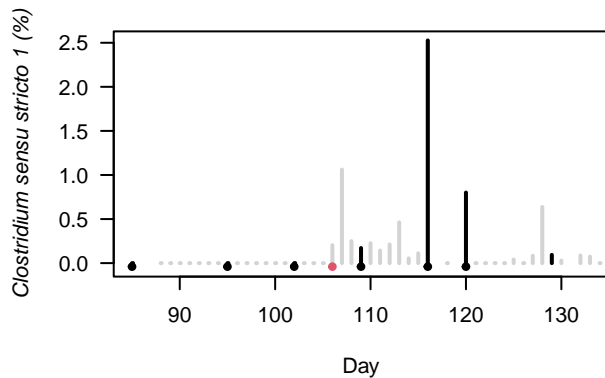

**Sample 5,  $p = 0.200$**

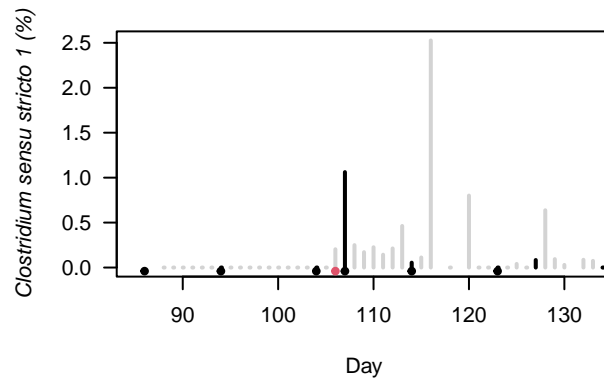

Supplement: Supplementary file 11 [file mmc11.pdf]
