# Supplementary material for: Sampling frequency matters: mapping of the healthy infants' gut microbiome during the first year of life
Source: Curr Res Microb Sci. 2025 Sep 9;9:100470. doi: 10.1016/j.crmicr.2025.100470 (PMC12466160; doi:10.1016/j.crmicr.2025.100470)
Supplement: Supplementary file 12 [file mmc12.pdf]

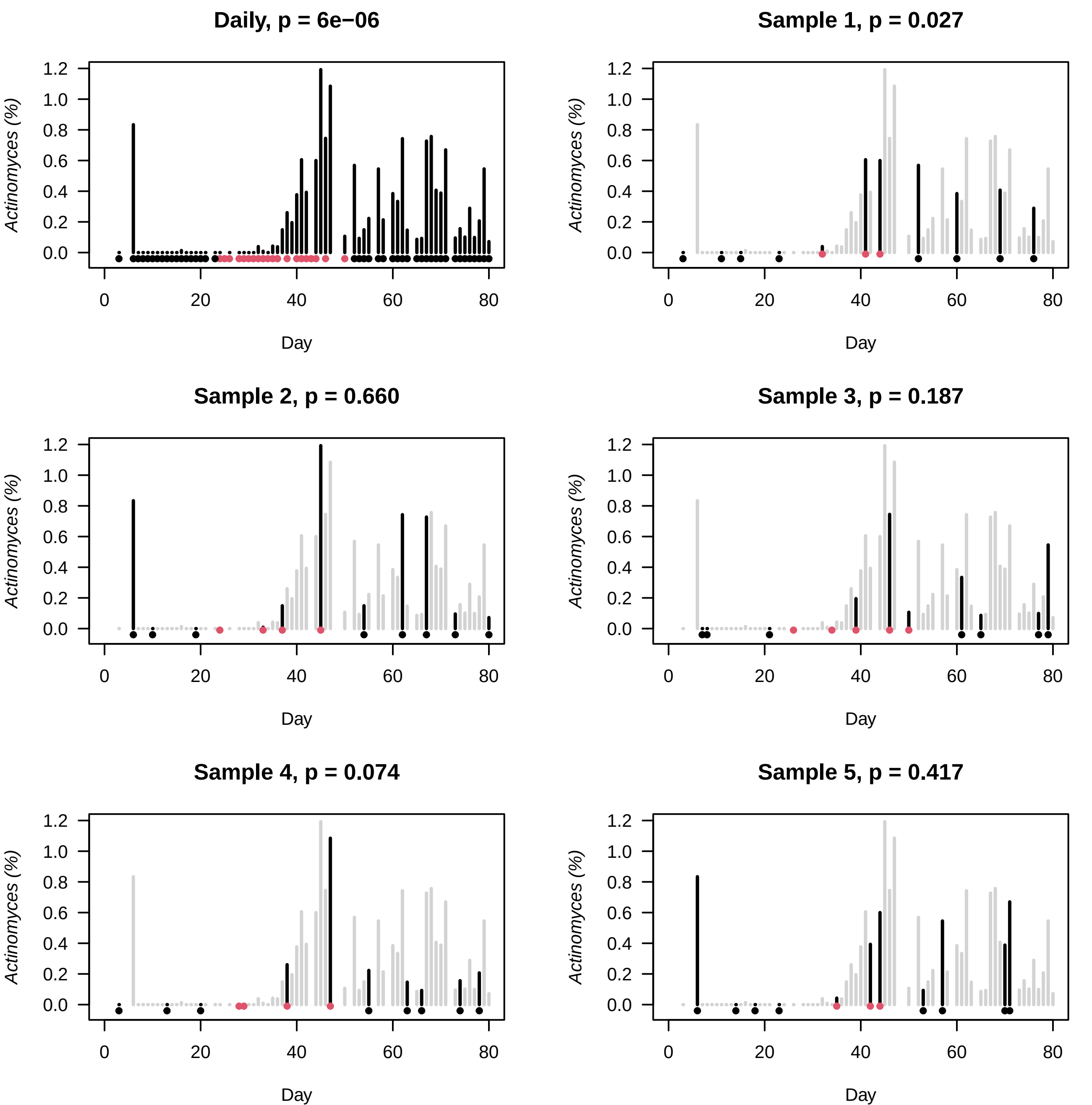

Daily, p = 6e−06
Sample 1, p = 0.027
Sample 2, p = 0.660
Sample 3, p = 0.187
Sample 4, p = 0.074
Sample 5, p = 0.417
Actinomyces (%)
Day
0.0
0.2
0.4
0.6
0.8
1.0
1.2
0
20
40
60
80

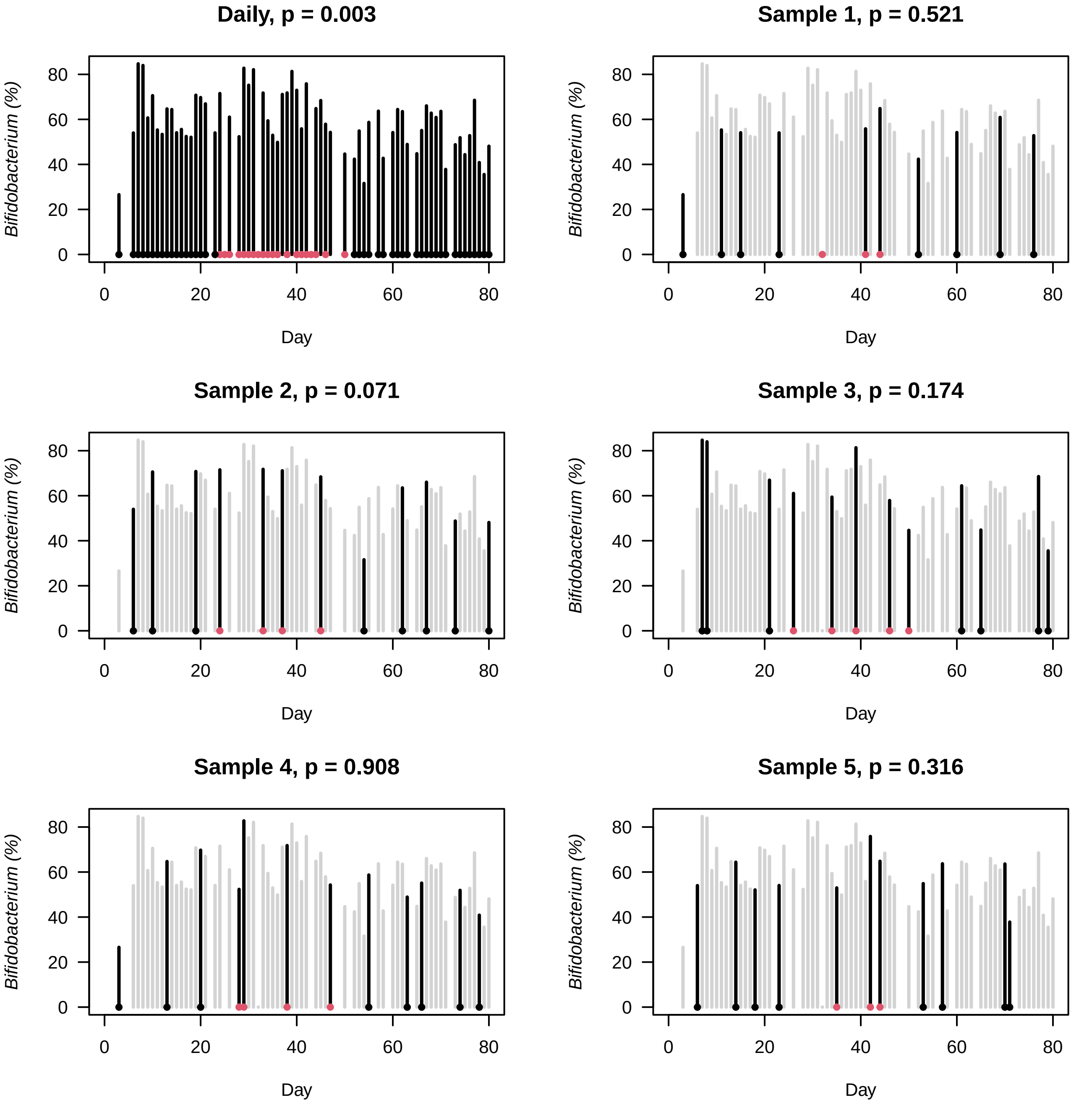

Daily, p = 0.003
Sample 1, p = 0.521
Sample 2, p = 0.071
Sample 3, p = 0.174
Sample 4, p = 0.908
Sample 5, p = 0.316
Bifidobacterium (%)
Day
0
20
40
60
80

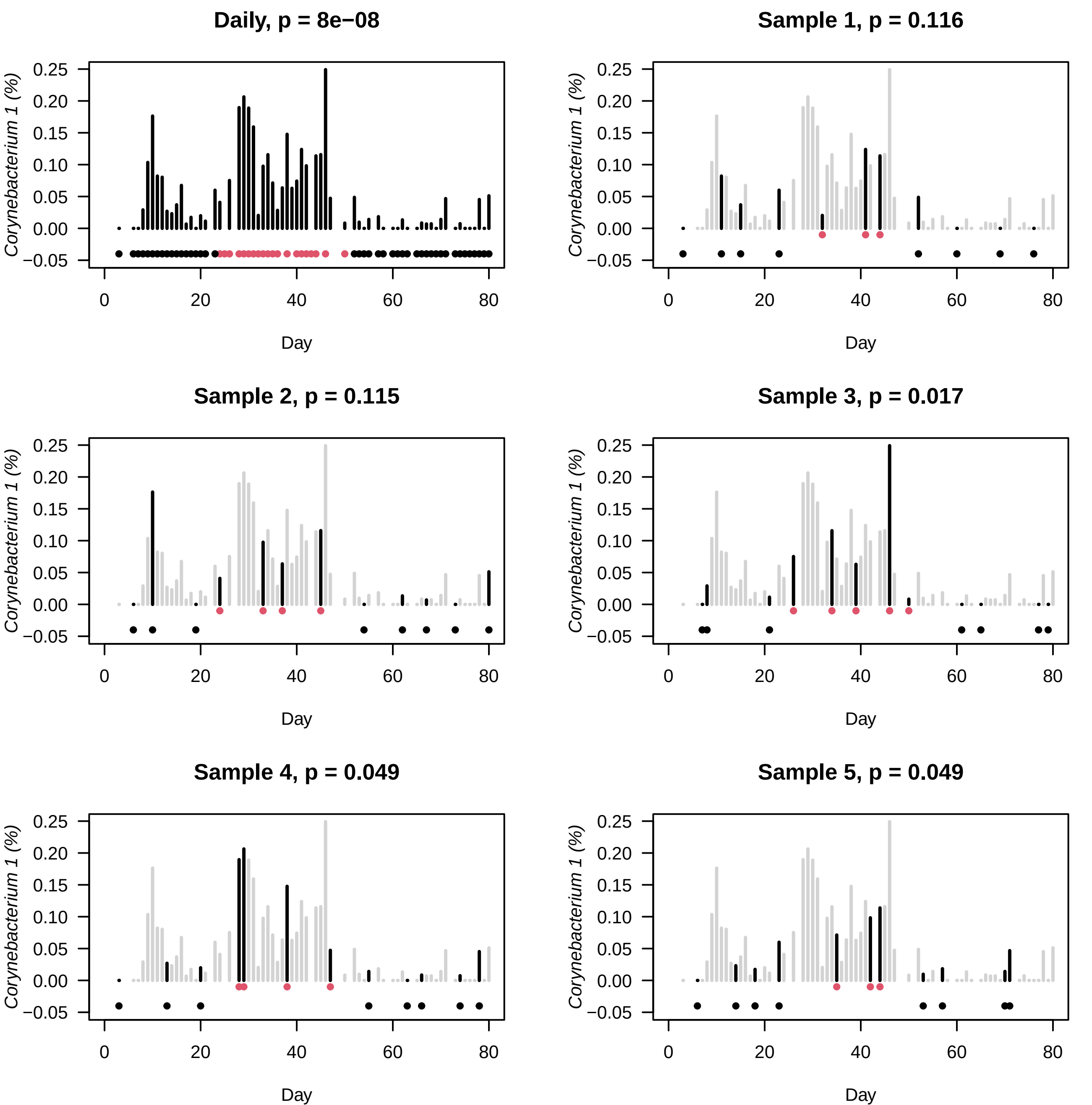
Daily, p = 8e−08
Sample 1, p = 0.116
Sample 2, p = 0.115
Sample 3, p = 0.017
Sample 4, p = 0.049
Sample 5, p = 0.049
Corynebacterium 1 (%)
Day
0.25
0.20
0.15
0.10
0.05
0.00
−0.05
0
20
40
60
80

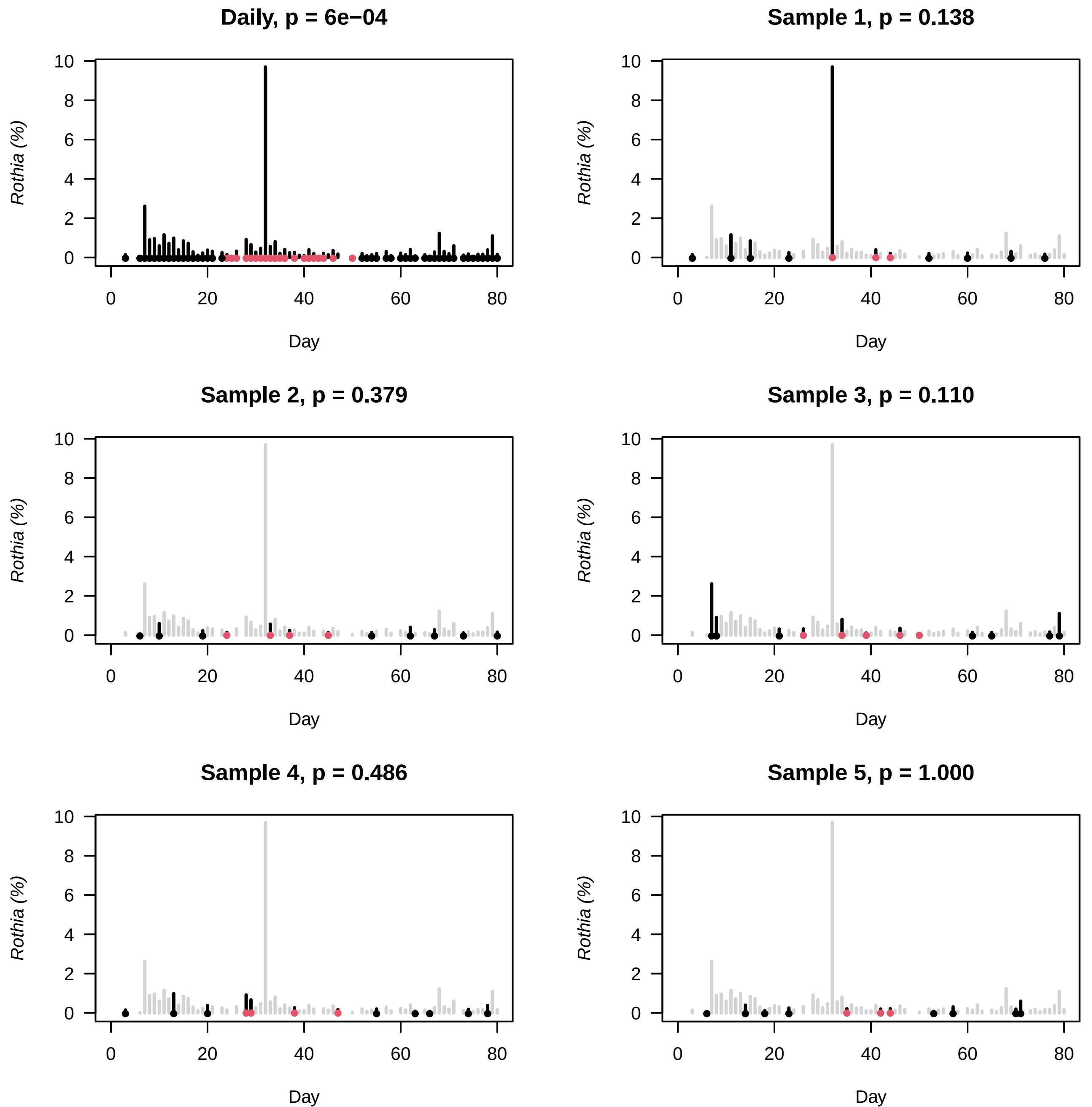

Daily, p = 6e−04
Sample 1, p = 0.138
Sample 2, p = 0.379
Sample 3, p = 0.110
Sample 4, p = 0.486
Sample 5, p = 1.000
Rothia (%)
Day
0
2
4
6
8
10
0
20
40
60
80

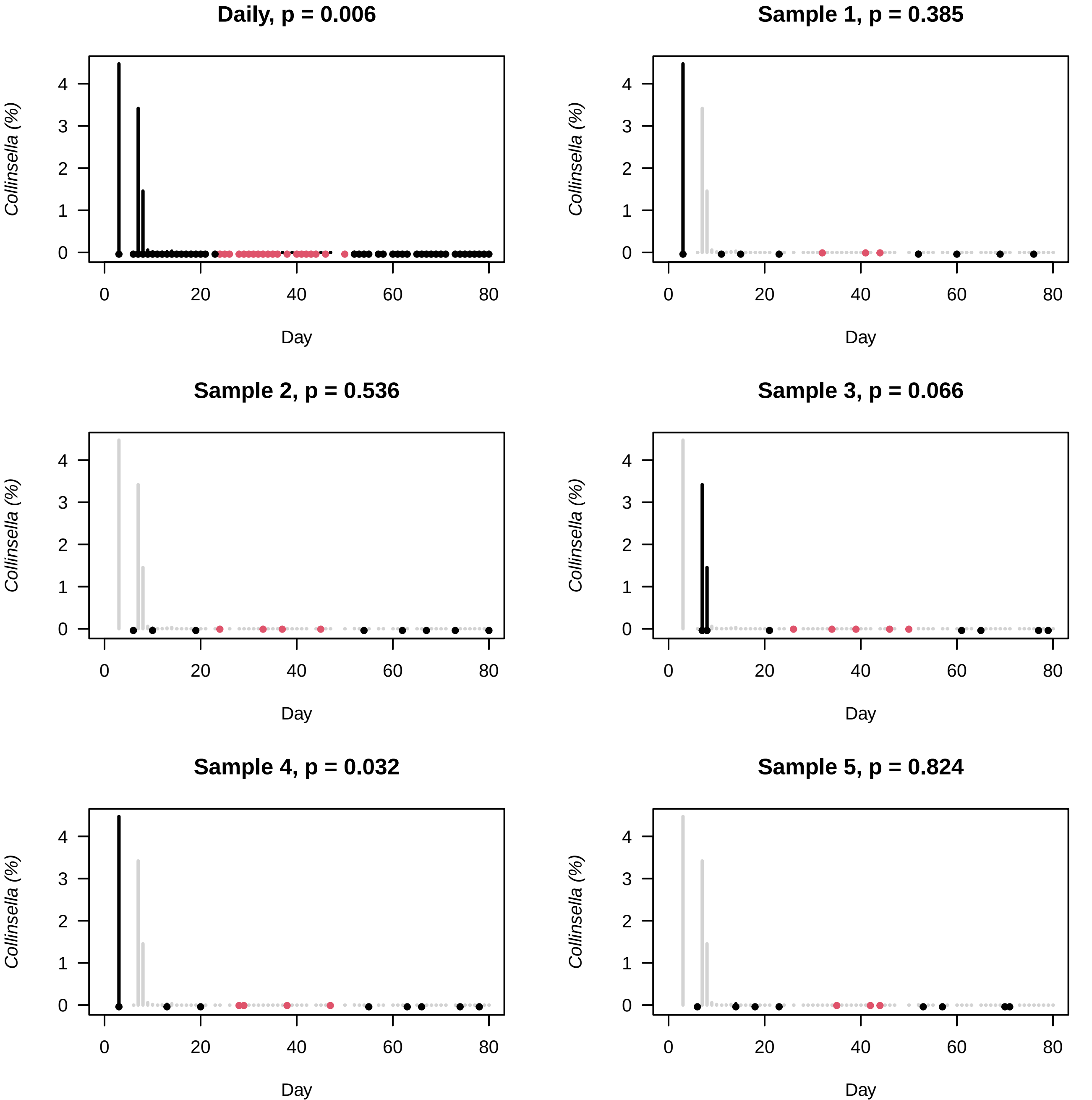

Daily, p = 0.006
Sample 1, p = 0.385
Sample 2, p = 0.536
Sample 3, p = 0.066
Sample 4, p = 0.032
Sample 5, p = 0.824
Collinsella (%)
Day
0
1
2
3
4
0
20
40
60
80

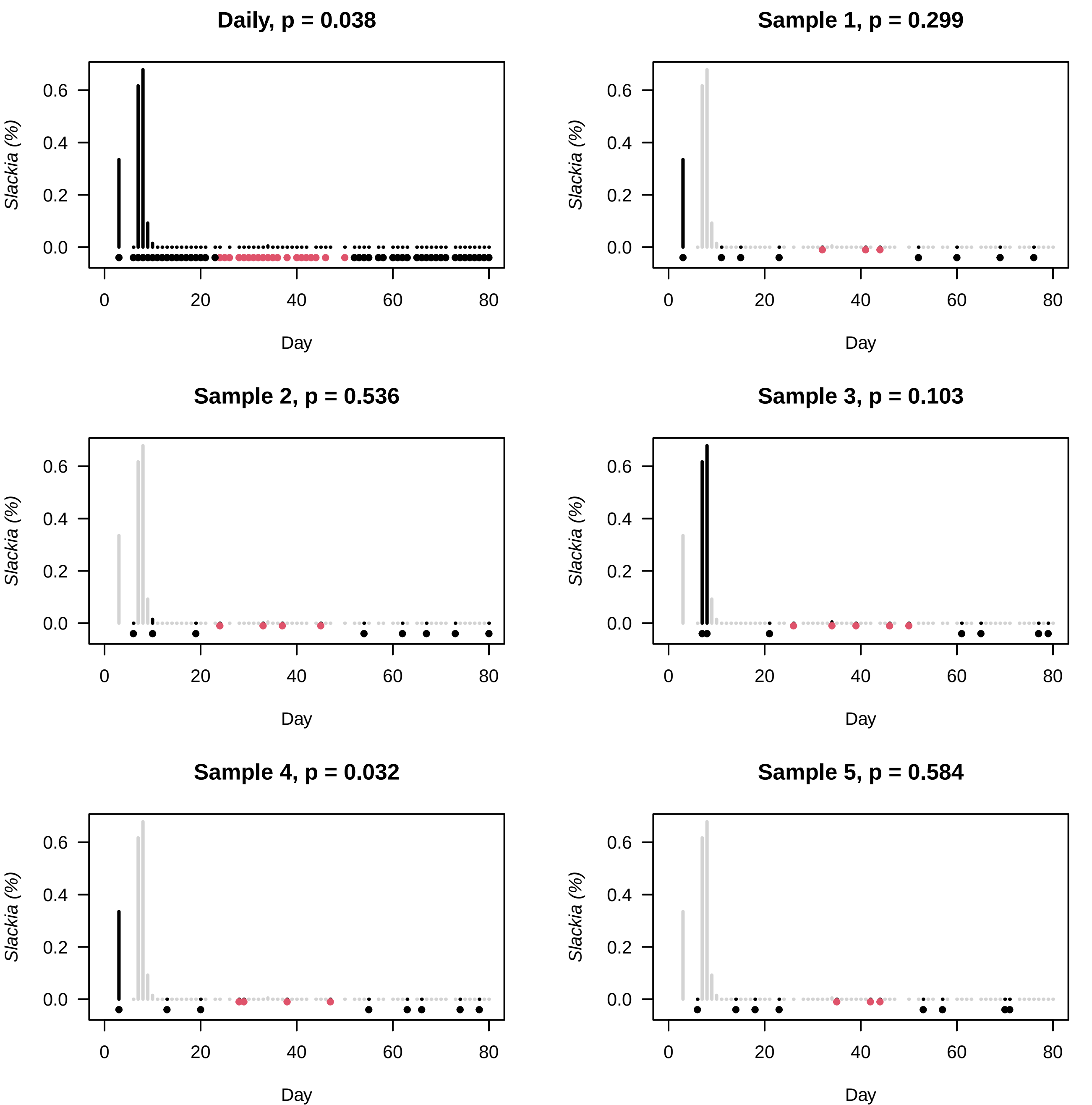
Daily, p = 0.038
Sample 1, p = 0.299
Sample 2, p = 0.536
Sample 3, p = 0.103
Sample 4, p = 0.032
Sample 5, p = 0.584
Slackia (%)
Day
0.0
0.2
0.4
0.6
0
20
40
60
80

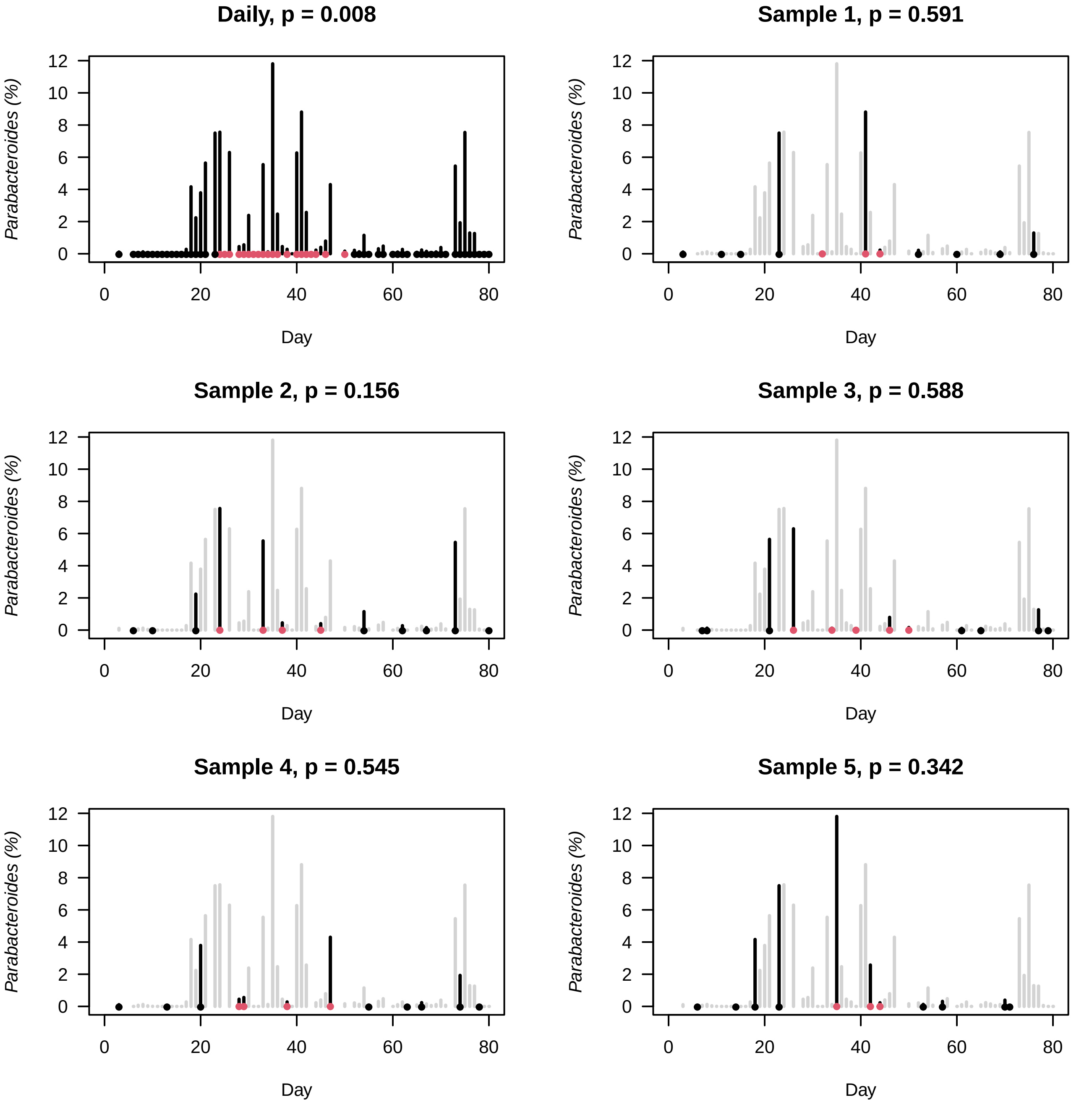

Daily, p = 0.008
Sample 1, p = 0.591
Sample 2, p = 0.156
Sample 3, p = 0.588
Sample 4, p = 0.545
Sample 5, p = 0.342
Parabacteroides (%)
Day

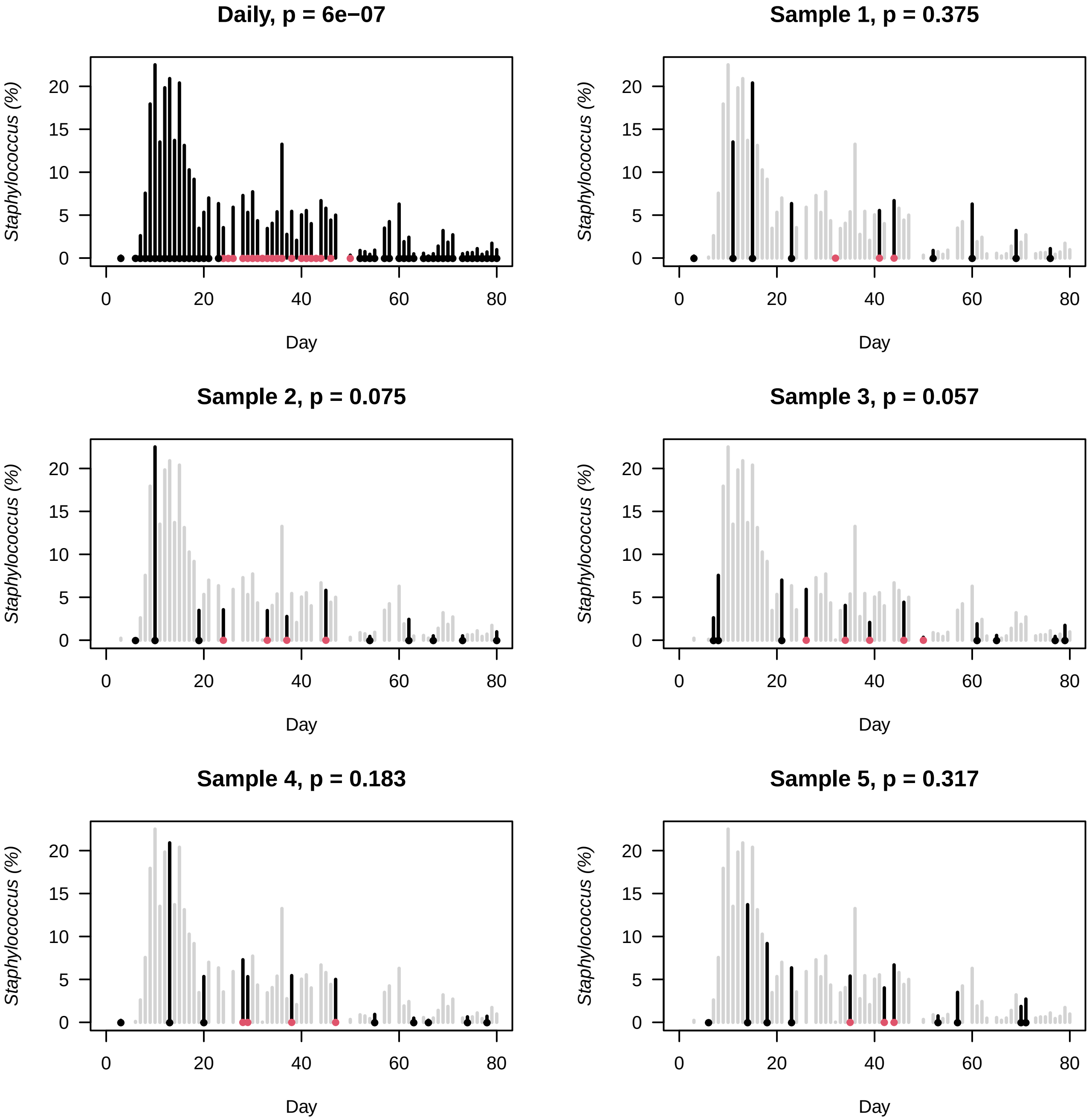

Daily, p = 6e−07
Sample 1, p = 0.375
Sample 2, p = 0.075
Sample 3, p = 0.057
Sample 4, p = 0.183
Sample 5, p = 0.317
Staphylococcus (%)
Day

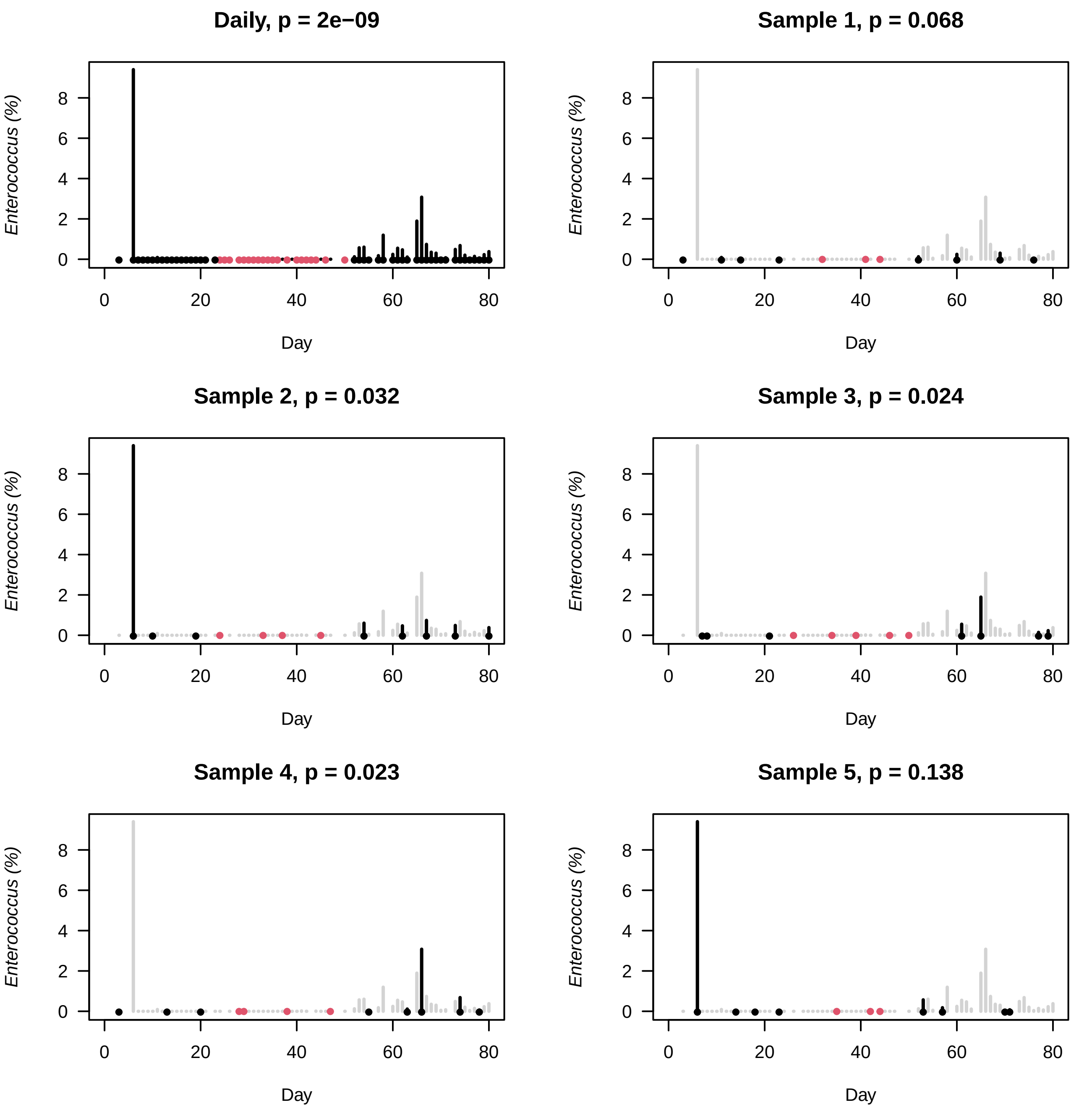

Daily, p = 2e−09
Sample 1, p = 0.068
Sample 2, p = 0.032
Sample 3, p = 0.024
Sample 4, p = 0.023
Sample 5, p = 0.138
Enterococcus (%)
Day
0
2
4
6
8
0
20
40
60
80

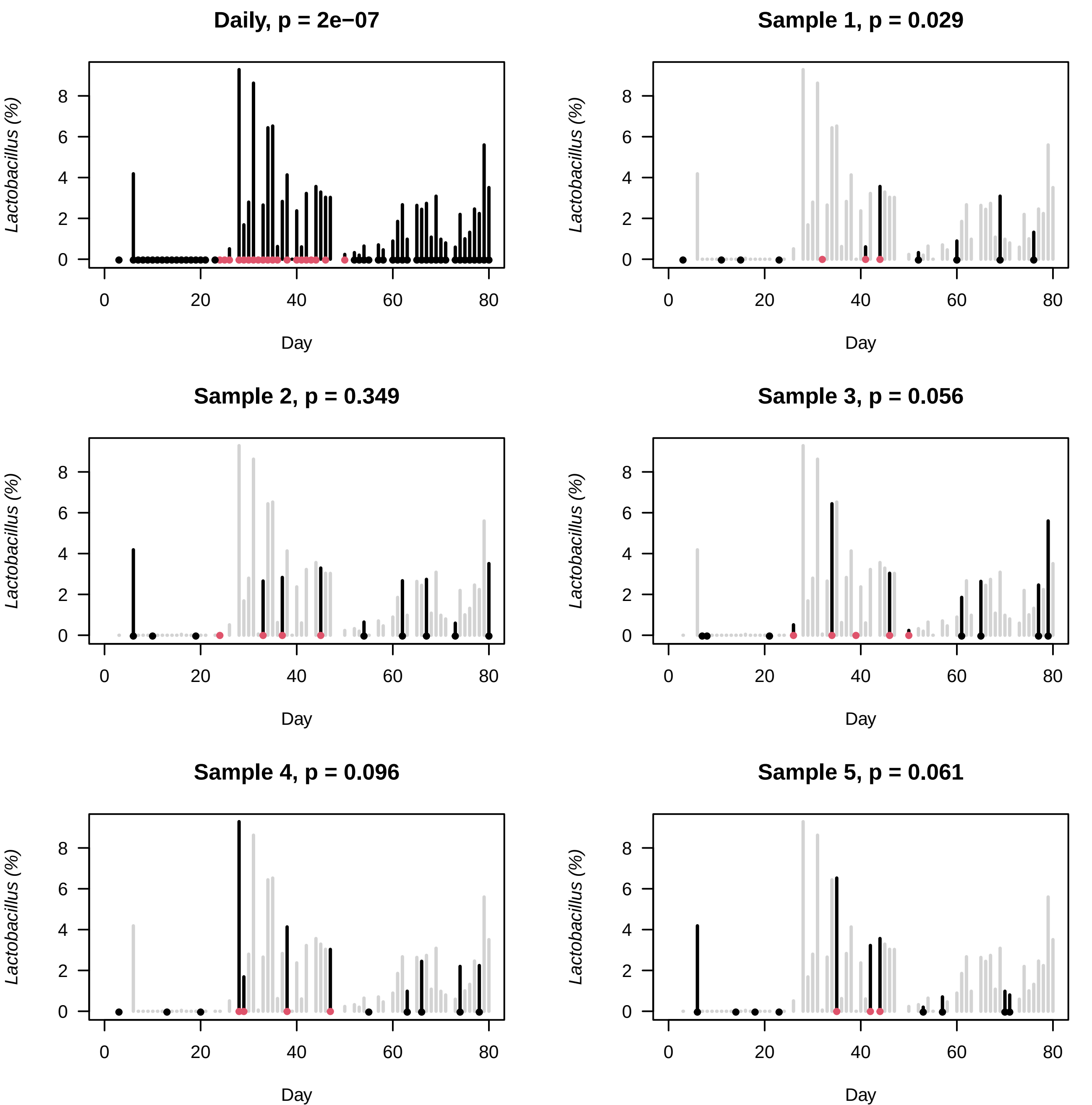

Daily, p = 2e−07
Sample 1, p = 0.029
Sample 2, p = 0.349
Sample 3, p = 0.056
Sample 4, p = 0.096
Sample 5, p = 0.061
Lactobacillus (%)
Day
0
20
40
60
80
0
2
4
6
8

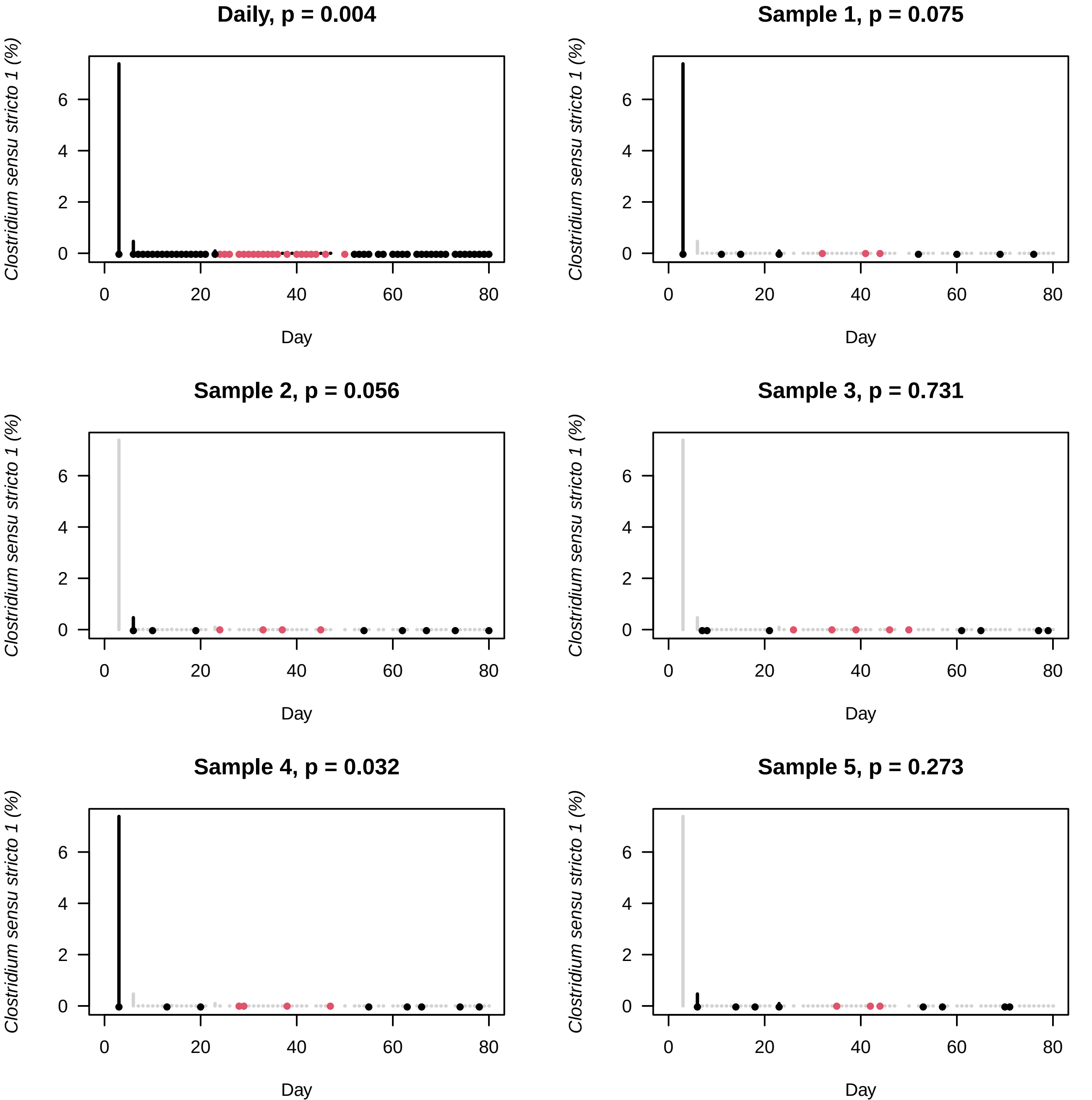

Daily, p = 0.004
Sample 1, p = 0.075
Sample 2, p = 0.056
Sample 3, p = 0.731
Sample 4, p = 0.032
Sample 5, p = 0.273
Clostridium sensu stricto 1 (%)
Day
0
20
40
60
80
0
2
4
6

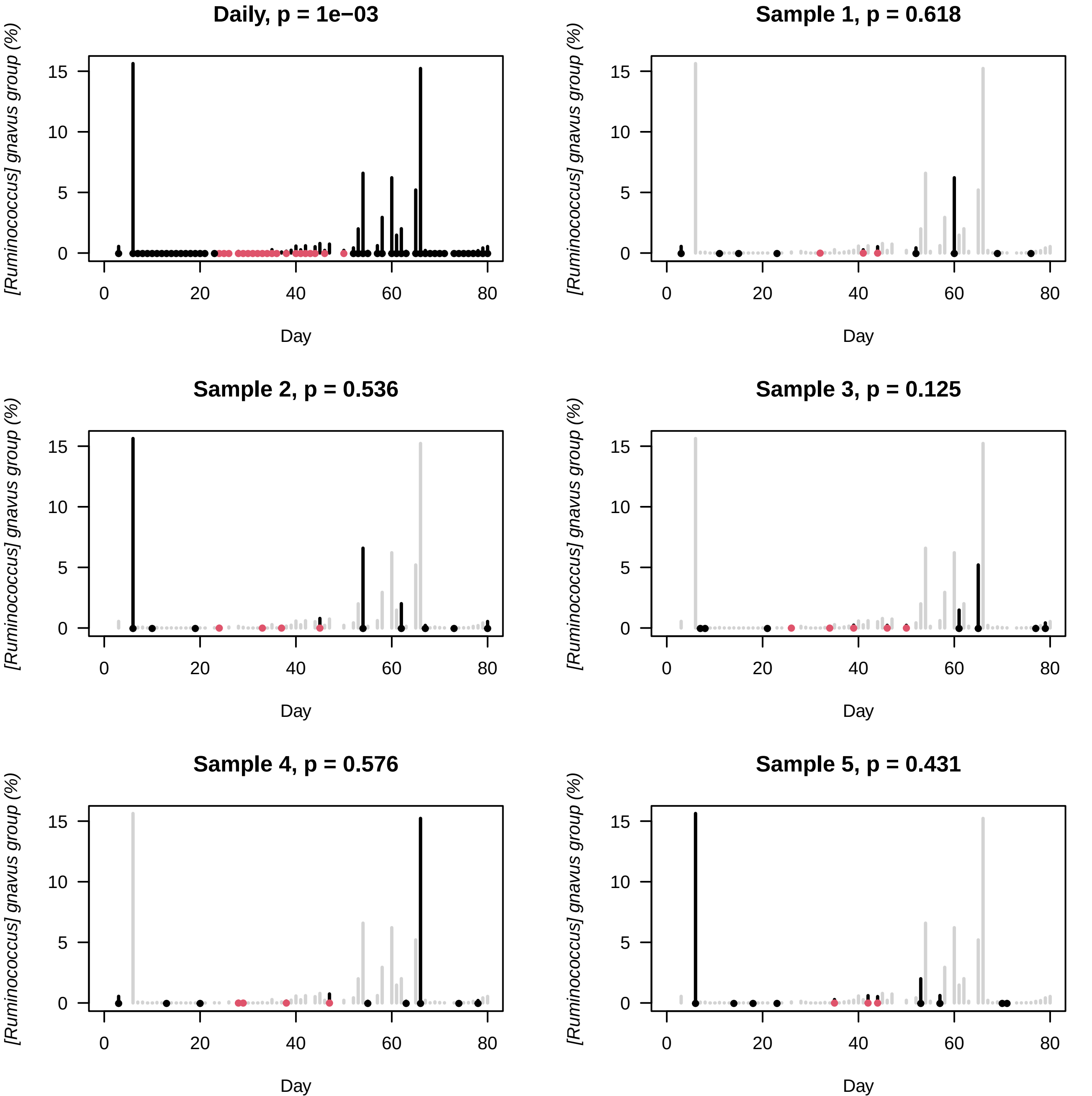

Daily, p = 1e−03
Sample 1, p = 0.618
Sample 2, p = 0.536
Sample 3, p = 0.125
Sample 4, p = 0.576
Sample 5, p = 0.431
[Ruminococcus] gnavus group (%)
Day
0
5
10
15
20
40
60
80

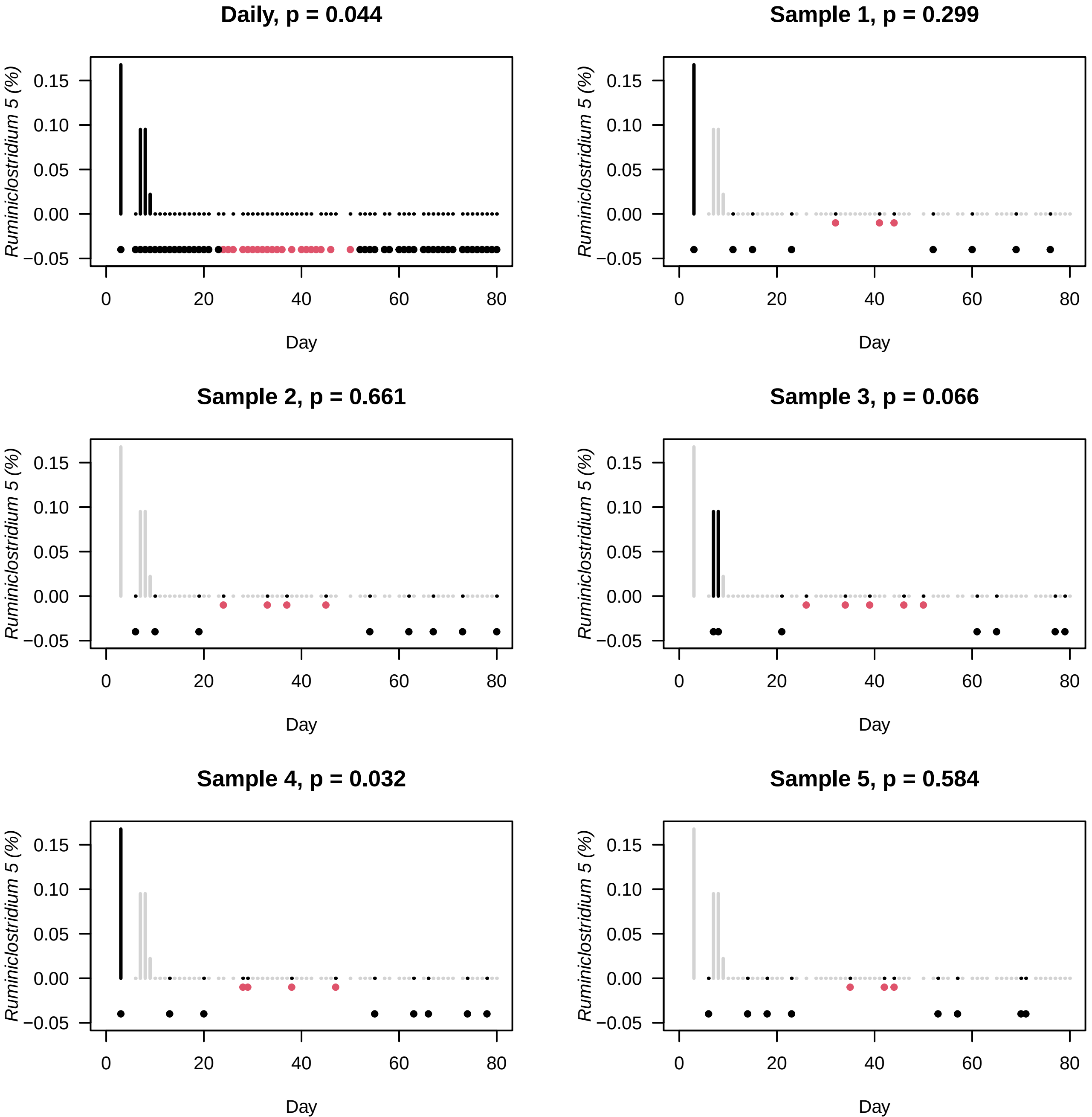
Daily, p = 0.044
Sample 1, p = 0.299
Sample 2, p = 0.661
Sample 3, p = 0.066
Sample 4, p = 0.032
Sample 5, p = 0.584
Ruminiclostridium 5 (%)
0.15
0.10
0.05
0.00
−0.05
0
20
40
60
80
Day

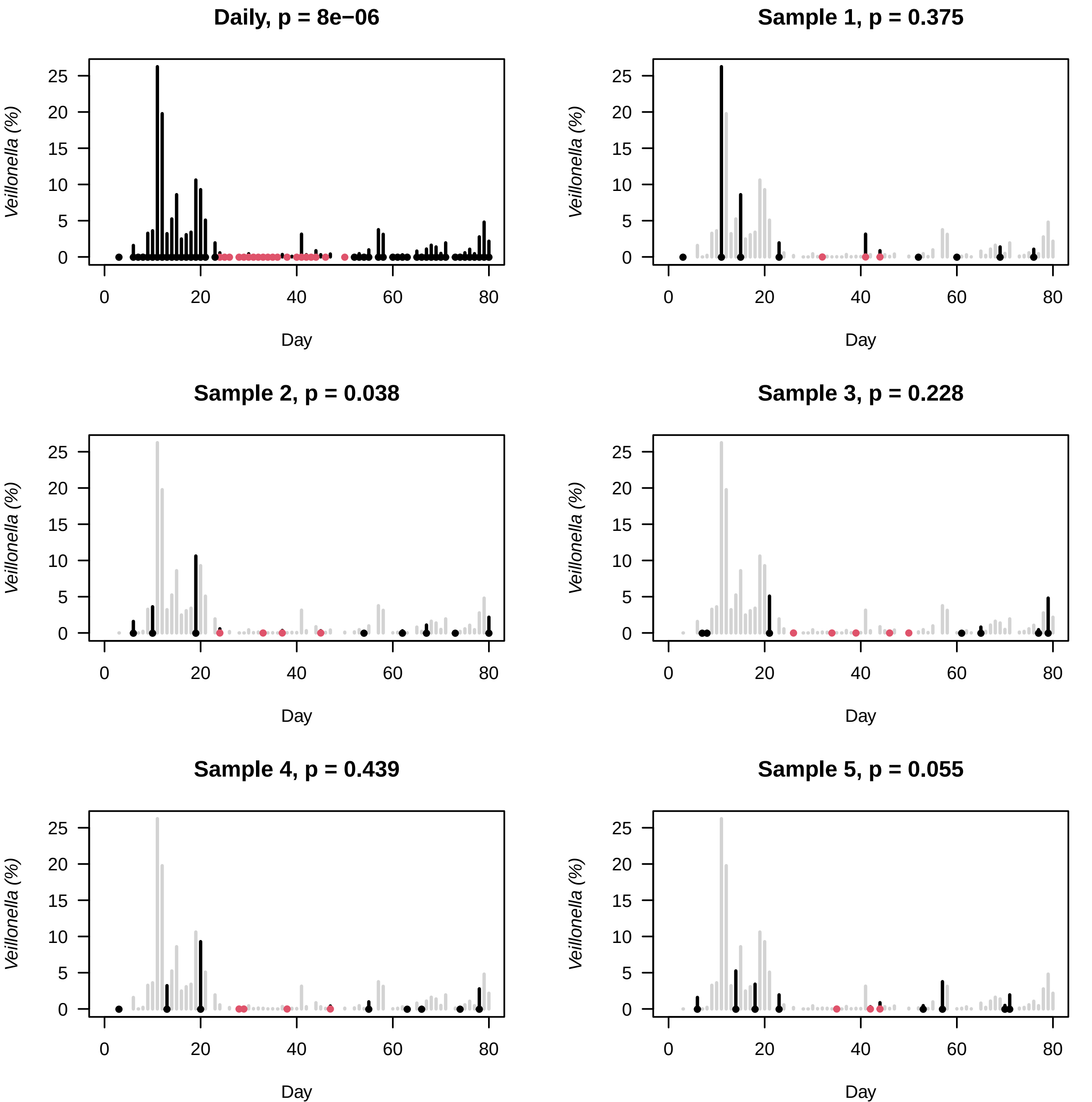

Daily, p = 8e−06
Sample 1, p = 0.375
Sample 2, p = 0.038
Sample 3, p = 0.228
Sample 4, p = 0.439
Sample 5, p = 0.055
Veillonella (%)
Day
0
5
10
15
20
25
0
20
40
60
80

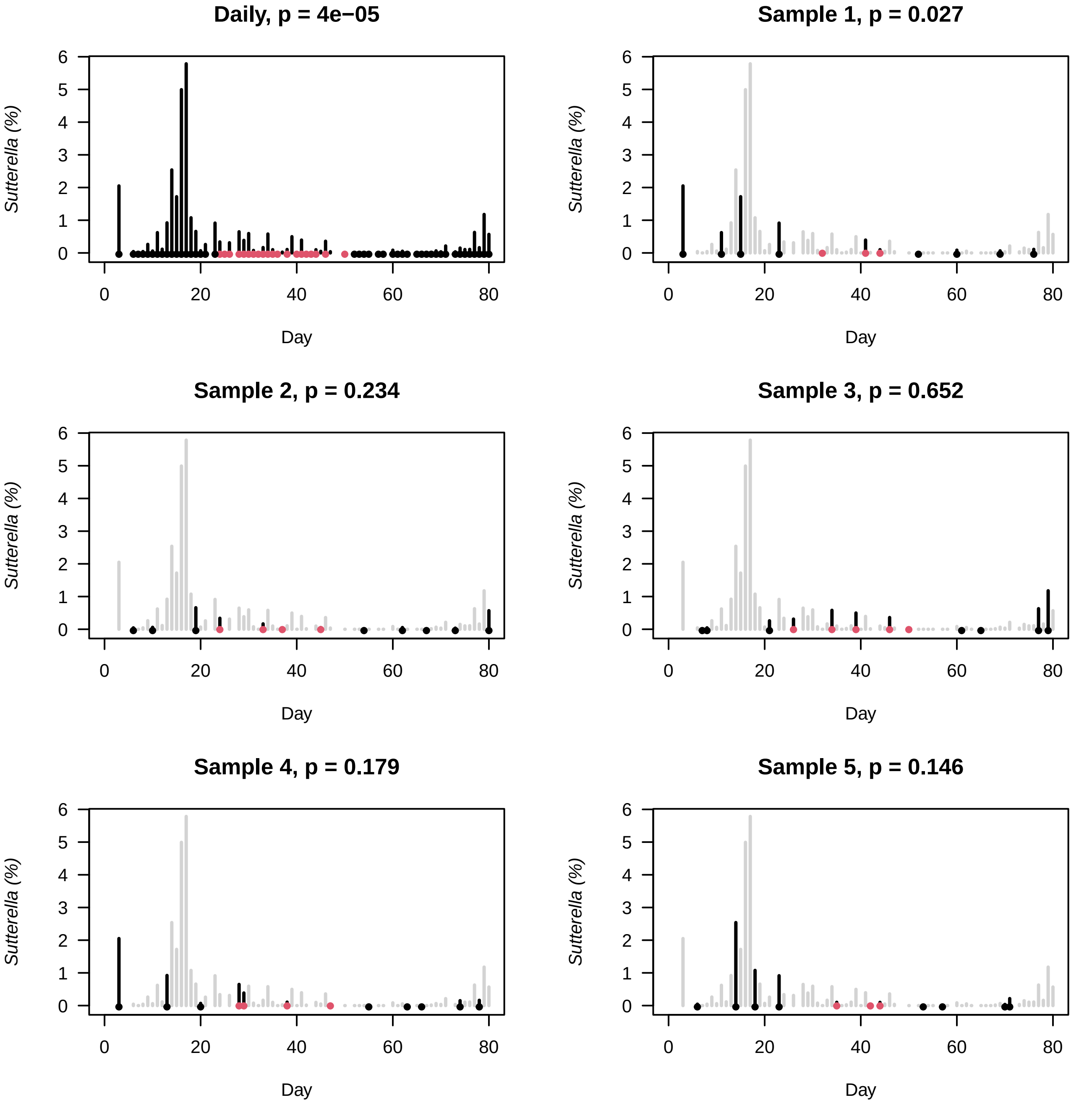

Daily, p = 4e−05
Sample 1, p = 0.027
Sample 2, p = 0.234
Sample 3, p = 0.652
Sample 4, p = 0.179
Sample 5, p = 0.146
Sutterella (%)
Day
0
1
2
3
4
5
6
0
20
40
60
80

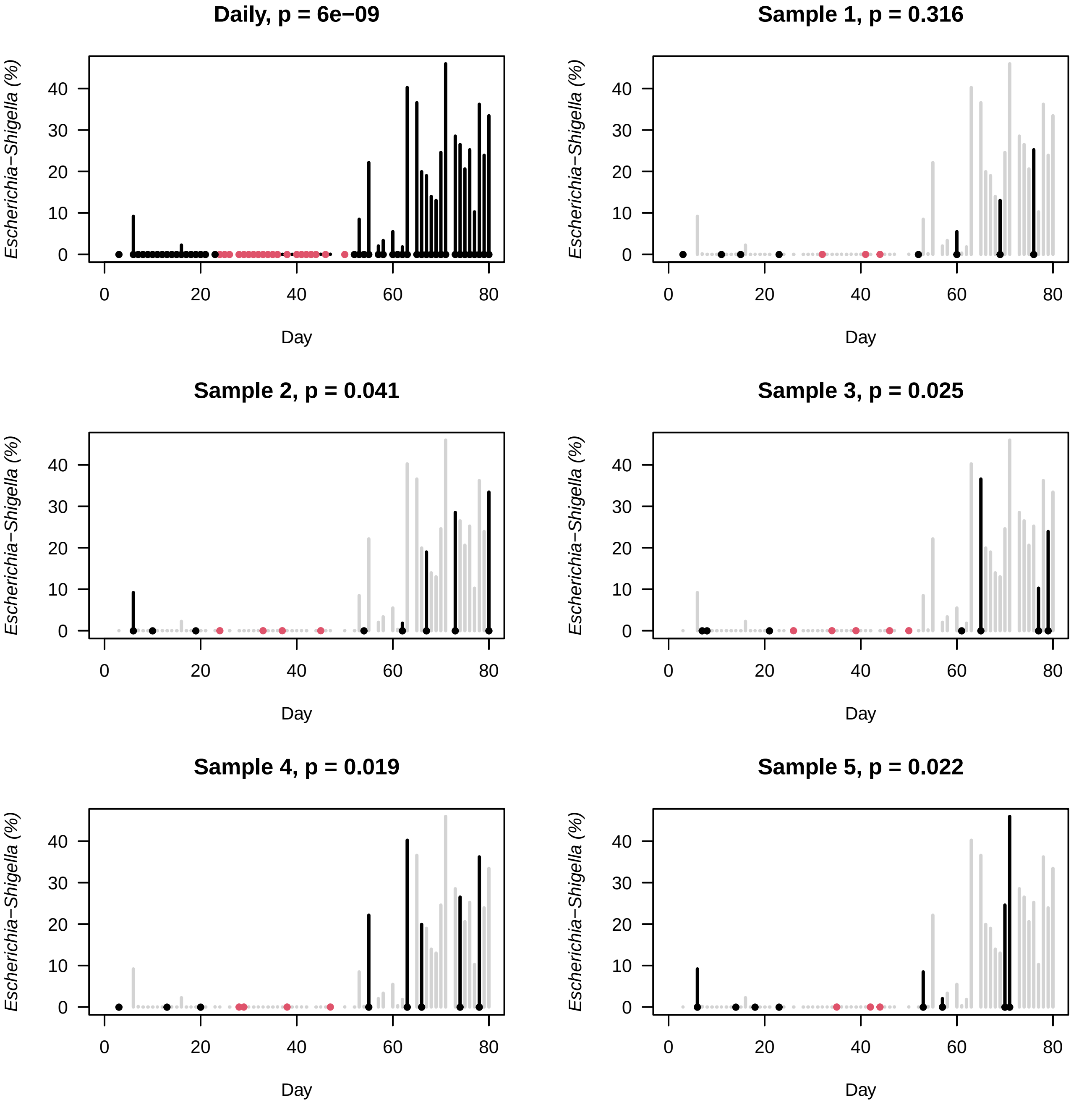
Daily, p = 6e−09
Sample 1, p = 0.316
Sample 2, p = 0.041
Sample 3, p = 0.025
Sample 4, p = 0.019
Sample 5, p = 0.022
Escherichia−Shigella (%)
Day
0
10
20
30
40
60
80

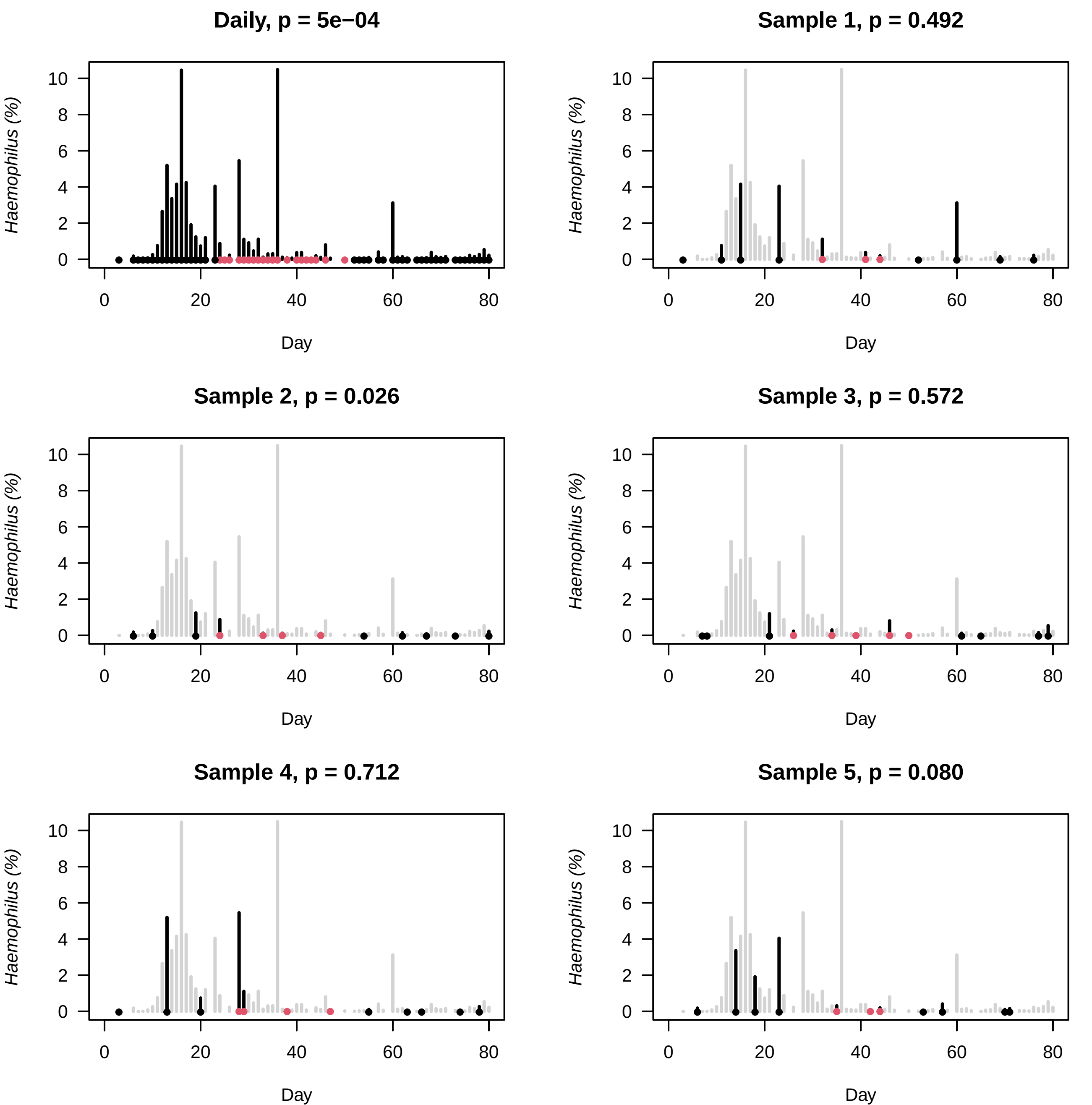

Daily, p = 5e−04
Sample 1, p = 0.492
Sample 2, p = 0.026
Sample 3, p = 0.572
Sample 4, p = 0.712
Sample 5, p = 0.080
Haemophilus (%)
Day
0
2
4
6
8
10
0
20
40
60
80
