# Supplementary material for: Sampling frequency matters: mapping of the healthy infants' gut microbiome during the first year of life
Source: Curr Res Microb Sci. 2025 Sep 9;9:100470. doi: 10.1016/j.crmicr.2025.100470 (PMC12466160; doi:10.1016/j.crmicr.2025.100470)
Supplement: Supplementary file 14 [file mmc14.docx]

**Table S2. List of primers and the length of PCR products**

| **Primer name** | | **5´-sequence-3´** | **Reference of primers** |  |
| --- | --- | --- | --- | --- |
| **EMP16S-1** | F | TCGTCGGCAGCGTCAGATGTGTATAAGAGACAGAGCCTTCGTCGCGTGTGYCAGCMGCCGCGGTAA | 16S Metagenomic sequencing Library  Preparation protocol; Illumina, USA (EMP 515-806) | |
|  | R | GTCTCGTGGGCTCGGAGATGTGTATAAGAGACAGCCTAACGGTCCACCGGACTACNVGGGTWTCTAAT |  |  |
| **EMP16S-2** | F | TCGTCGGCAGCGTCAGATGTGTATAAGAGACAGTCCATACCGGAAGTGTGYCAGCMGCCGCGGTAA |  |  |
|  | R | GTCTCGTGGGCTCGGAGATGTGTATAAGAGACAGCGCGCCTTAAACCCGGACTACNVGGGTWTCTAAT |  |  |
| **EMP16S-3** | F | TCGTCGGCAGCGTCAGATGTGTATAAGAGACAGAGCCCTGCTACAGTGTGYCAGCMGCCGCGGTAA |  |  |
|  | R | GTCTCGTGGGCTCGGAGATGTGTATAAGAGACAGTATGGTACCCAGCCGGACTACNVGGGTWTCTAAT |  |  |
| **EMP16S-4** | F | TCGTCGGCAGCGTCAGATGTGTATAAGAGACAGTGAGACCCTACAGTGTGYCAGCMGCCGCGGTAA |  |  |
|  | R | GTCTCGTGGGCTCGGAGATGTGTATAAGAGACAGGCCTCTACGTCGCCGGACTACNVGGGTWTCTAAT |  |  |
| **EMP16S-5** | F | TCGTCGGCAGCGTCAGATGTGTATAAGAGACAGACTTGGTGTAAGGTGTGYCAGCMGCCGCGGTAA |  |  |
|  | R | GTCTCGTGGGCTCGGAGATGTGTATAAGAGACAGACTACTGAGGATCCGGACTACNVGGGTWTCTAAT |  |  |
| **EMP16S-6** | F | TCGTCGGCAGCGTCAGATGTGTATAAGAGACAGATTACGTATCATGTGTGYCAGCMGCCGCGGTAA |  |  |
|  | R | GTCTCGTGGGCTCGGAGATGTGTATAAGAGACAGAATTCACCTCCTCCGGACTACNVGGGTWTCTAAT |  |  |
| **EMP16S-7** | F | TCGTCGGCAGCGTCAGATGTGTATAAGAGACAGCACGCAGTCTACGTGTGYCAGCMGCCGCGGTAA |  |  |
|  | R | GTCTCGTGGGCTCGGAGATGTGTATAAGAGACAGCGTATAAATGCGCCGGACTACNVGGGTWTCTAAT |  |  |
| **EMP16S-8** | F | TCGTCGGCAGCGTCAGATGTGTATAAGAGACAGTGTGCACGCCATGTGTGYCAGCMGCCGCGGTAA |  |  |
|  | R | GTCTCGTGGGCTCGGAGATGTGTATAAGAGACAGATGCTGCAACACCCGGACTACNVGGGTWTCTAAT |  |  |
| **EMP16S-9** | F | TCGTCGGCAGCGTCAGATGTGTATAAGAGACAGCCGGACAAGAAGGTGTGYCAGCMGCCGCGGTAA |  |  |
|  | R | GTCTCGTGGGCTCGGAGATGTGTATAAGAGACAGACTCGCTCGCTGCCGGACTACNVGGGTWTCTAAT |  |  |
| **EMP16S-10** | F | TCGTCGGCAGCGTCAGATGTGTATAAGAGACAGTTGCTGGACGCTGTGTGYCAGCMGCCGCGGTAA |  |  |
|  | R | GTCTCGTGGGCTCGGAGATGTGTATAAGAGACAGTTCCTTAGTAGTCCGGACTACNVGGGTWTCTAAT |  |  |
| **EMP16S-11** | F | TCGTCGGCAGCGTCAGATGTGTATAAGAGACAGTACTAACGCGGTGTGTGYCAGCMGCCGCGGTAA |  |  |
|  | R | GTCTCGTGGGCTCGGAGATGTGTATAAGAGACAGCGTCCGTATGAACCGGACTACNVGGGTWTCTAAT |  |  |
| **EMP16S-12** | F | TCGTCGGCAGCGTCAGATGTGTATAAGAGACAGGCGATCACACCTGTGTGYCAGCMGCCGCGGTAA |  |  |
|  | R | GTCTCGTGGGCTCGGAGATGTGTATAAGAGACAGACGTGAGGAACGCCGGACTACNVGGGTWTCTAAT |  |  |
| **EMP16S-13** | F | TCGTCGGCAGCGTCAGATGTGTATAAGAGACAGCAAACGCACTAAGTGTGYCAGCMGCCGCGGTAA |  |  |
|  | R | GTCTCGTGGGCTCGGAGATGTGTATAAGAGACAGGGTTGCCCTGTACCGGACTACNVGGGTWTCTAAT |  |  |
| **EMP16S-14** | F | TCGTCGGCAGCGTCAGATGTGTATAAGAGACAGGAAGAGGGTTGAGTGTGYCAGCMGCCGCGGTAA |  |  |
|  | R | GTCTCGTGGGCTCGGAGATGTGTATAAGAGACAGCATATAGCCCGACCGGACTACNVGGGTWTCTAAT |  |  |
| **EMP16S-15** | F | TCGTCGGCAGCGTCAGATGTGTATAAGAGACAGTGAGTGGTCTGTGTGTGYCAGCMGCCGCGGTAA |  |  |
|  | R | GTCTCGTGGGCTCGGAGATGTGTATAAGAGACAGGCCTATGAGATCCCGGACTACNVGGGTWTCTAAT |  |  |
